# Remote monitoring of rheumatoid arthritis (REMORA): study protocol for a stepped wedge cluster randomized trial and process evaluation of an integrated symptom tracking intervention

**DOI:** 10.1186/s13063-024-08497-9

**Published:** 2024-10-15

**Authors:** Sabine N. van der Veer, Deb Griffiths-Jones, Matthew Parkes, Katie L. Druce, Paul Amlani-Hatcher, Christopher J. Armitage, Nicholas Bansback, Peter Bower, Dawn Dowding, Benjamin Ellis, Jill Firth, Sean Gavan, Elaine Mackey, Caroline Sanders, Charlotte A. Sharp, Karen Staniland, William G. Dixon, the REMORA team

**Affiliations:** 1 Centre for Health Informatics, Division of Informatics, Imaging and Data Science, University of Manchester, Manchester Academic Health Science Centre, Vaughan House, Portsmouth Street, Manchester, M13 9GB UK; 2 Centre for Epidemiology Versus Arthritis, Division of Musculoskeletal and Dermatological Sciences, The University of Manchester, Manchester Academic Health Science Centre, Manchester, UK; 3 Centre for Biostatistics, Division of Population Health, Health Services Research & Primary Care, School of Health Sciences, The University of Manchester, Manchester, UK; 4 NIHR Manchester Biomedical Research Centre, Manchester University NHS Foundation Trust, Manchester Academic Health Science Centre (MAHSC), Manchester, UK; 5 Manchester Centre for Health Psychology, Division of Psychology and Mental Health, The University of Manchester, Manchester Academic Health Science Centre, Manchester, UK; 6 NIHR Greater Manchester Patient Safety Research Collaboration, The University of Manchester, Manchester Academic Health Science Centre, Manchester, UK; 7 School of Population and Public Health, University of British Columbia, Vancouver, Canada; 8 Centre for Primary Care and Health Services Research, Division of Population Health, Health Services Research and Primary Care, The University of Manchester, Manchester Academic Health Science Centre, Manchester, UK; 9 Division of Nursing, Midwifery and Social Work, School of Health Sciences, Faculty of Biomedicine and Health, The University of Manchester, Manchester, UK; 10 Imperial College Healthcare NHS Trust, London, UK; 11 Pennine MSK Partnership, Integrated Care Centre, Oldham, UK; 12 Manchester Centre for Health Economics, Division of Population Health, Health Services Research and Primary Care, Manchester Academic Health Science Centre, The University of Manchester, Manchester, UK; 13 Kellgren Centre for Rheumatology, Manchester Royal Infirmary, Manchester University NHS Foundation Trust, Manchester, UK; 14 Rheumatology Department, Salford Royal Hospital, Northern Care Alliance NHS Foundation Trust, Salford, UK

**Keywords:** Mobile health, Patient-generated health data, Randomized controlled trial, Rheumatology, Signs and symptoms, Telemedicine

## Abstract

**Background:**

Management of rheumatoid arthritis (RA) relies on symptoms reported by patients during infrequent outpatient clinic visits. These reports are often incomplete and inaccurate due to poor recall, leading to suboptimal treatment decisions and outcomes. Asking people to track symptoms in-between visits and integrating the data into clinical pathways may improve this. However, knowledge on how to implement this into practice and its impact on services and outcomes remains scarce in RA. Therefore, we evaluate the comparative effectiveness and cost-effectiveness of integrated symptom tracking in people with RA over and above usual care, while generating insights on factors for successful implementation.

**Methods:**

In this superiority stepped wedge cluster-randomized controlled trial with continuous recruitment short exposure design, 16 rheumatology outpatient departments (clusters) recruit a total of 732 people with active RA. They initially offer clinic visits according to standard of care before switching in pairs to visits with integrated symptom tracking. Clusters switch in randomized order every 3 weeks. Integrated symptom tracking consists of (1) a mobile app for patients to track their symptoms daily and other RA aspects weekly/monthly, and (2) an interactive dashboard visualizing the app data, which healthcare professionals access from their electronic health record system. Clinic visits happen according to usual practice, with tracked symptom data only reviewed during visits. Our primary outcome is a difference in marginal mean disease activity score at 12 ± 3 months between standard of care and integrated symptom tracking, after accounting for baseline values, cluster, and other covariates. Secondary outcomes include patient-reported disease activity, quality of life and quality-adjusted life-years, medication/resource use, consultation and decision-making experience, self-management, and illness perception. We also conduct interviews and observations as part of a parallel process evaluation to gather information on implementation.

**Discussion:**

Our trial will generate high-quality evidence of comparative and cost-effectiveness of integrated symptom tracking compared to standard of care in people with RA, with our process evaluation delivering knowledge on successful implementation. This optimizes the chances of integrated symptom tracking being adopted more widely if we find it is (cost-) effective.

**Trial registration:**

Registered 4-Jun-2024 on https://www.isrctn.com/, ISRCTN51539448.

**Trial open science framework repository:**

https://osf.io/sj9ha/.

**Supplementary Information:**

The online version contains supplementary material available at 10.1186/s13063-024-08497-9.

## Introduction

One in three people in the United Kingdom (UK) and in other countries live with at least one long-term condition, collectively accounting for a significant part of all healthcare expenditure [1–3]. One such long-term condition is rheumatoid arthritis (RA): a chronic immune-mediated inflammatory disease that causes joint pain, swelling, stiffness and associated disability. RA affects many people worldwide [4], including just below 1% of the UK population [5].

Long-term conditions, such as RA, are commonly managed jointly by general practitioners and hospital specialists, where the latter decide on treatment based on infrequent outpatient clinic visits that happen once every 3–12 months [6, 7]. During these visits, assessment of disease activity relies on patient descriptions of symptoms and flares. However, answering the hospital specialist’s question ‘How have you been since your last visit?’ is challenging for patients because it asks them to recall flares and summarize fluctuating symptoms over an extended period [8]. This results in incomplete and inaccurate information [9, 10], which may lead to suboptimal treatment decisions and ultimately to worse outcomes [11].

Asking people to track their symptoms between clinic visits has the potential to provide a clearer picture of disease. In the context of RA trials, daily symptom tracking confirmed its benefits of reducing recall error and enabling more robust evaluation of treatment response [12]. Information on tracked symptoms, if made available during visits, could inform better shared decision-making [13, 14]. For example, by revealing flares that would otherwise remain undetected, or by providing a completer and more accurate picture of response to changes in medication [15, 16]. Better decision-making may, in turn, lead to better long-term outcomes, as well as making patients feel more empowered and improving their satisfaction with care [17]. Lastly, symptom tracking may enhance people’s confidence and ability to self-manage their condition [13, 18], which could reduce burden on healthcare services [19].

The interest in integrating electronic patient-generated health data, such as tracked symptoms, into clinical pathways is growing, with national healthcare strategies recognizing its potential to transform outpatient services and make them more person-centred [20–22]. To date, however, evidence of its impact on services and outcomes and knowledge on factors affecting its successful implementation remain scarce in RA and other musculoskeletal diseases [23–25]. Previous studies in RA were not randomized, were conducted in a single centre, recruited a small and/or highly selected sample, used lower-tech interventions (e.g. SMS text messages), collected patient-generated health data infrequently (e.g. monthly), did not integrate it into electronic health records and/or did not assess the impact on clinical outcomes or patient experience [25–31].

After completing a successful proof-of-concept study [15] and mixed-methods feasibility trial [32], we are therefore undertaking a stepped wedge cluster randomized controlled trial called REmote MOnitoring in Rheumatoid Arthritis 2 (REMORA2). It evaluates the comparative effectiveness and cost-effectiveness of integrated symptom tracking in people living with RA over and above usual care, while also generating insights into its impact on shared decision-making and into factors that influence its successful implementation in clinical practice. The current protocol details our plans for the REMORA2 trial and follows the Standard Protocol Items: Recommendations for Interventional Trials (SPIRIT) outcomes 2022 extension reporting guidance [33] (see Additional file 1 for completed checklist).

## Objectives

Our stepped wedge cluster randomized controlled trial aims to evaluate if integrating frequent patient-reported symptom tracking into clinical pathways and systems improves care and outcomes for people living with long-term conditions, using RA as an exemplar. A parallel process evaluation [34] aims to generate knowledge on how to successfully implement electronic patient-generated health data into clinical practice, including the necessary change of workflows and patients’ and healthcare professionals’ behaviours.

### Primary objective

The primary objective of the trial (objective 1) is to evaluate the effect of clinic visits with integrated symptom tracking compared with standard of care on disease activity in people living with RA attending rheumatology consultations in outpatient hospital care settings at 12 months’ follow-up.

### Secondary objectives

The secondary objectives of the trial are to:

2. Evaluate the effect of clinic visits with integrated symptom tracking, compared with standard of care, on:
  - Secondary quantitative indicators of disease activity and impact at 12 months’ follow-up.
  - Shared decision-making, self-management and people’s consultation experience, using mixed methods.
3. Identify barriers to behaviour change, intervention uptake, and wider implementation into clinical practice, and ways to address these barriers.
4. Evaluate the incremental cost-effectiveness of integrated symptom tracking in RA compared with standard of care.
5. Explore possible mechanisms that may explain any observed improvement in disease activity (or lack thereof) in relation to integrated symptom tracking, using mixed methods.

### Tertiary objectives

Depending on available time and resources within the study team, we may address tertiary objectives by analysing data collected for addressing the primary and secondary objectives. Potential examples include examining the representativeness and diversity of study participants compared to the wider RA population, exploring longitudinal trajectories of symptoms in response to treatment, and identifying factors associated with patients’ engagement with integrated symptom tracking. A final set of tertiary objectives will be determined at a later stage and will therefore not be described further in this protocol.

## Methods

### Trial design

**Fig. 1:**
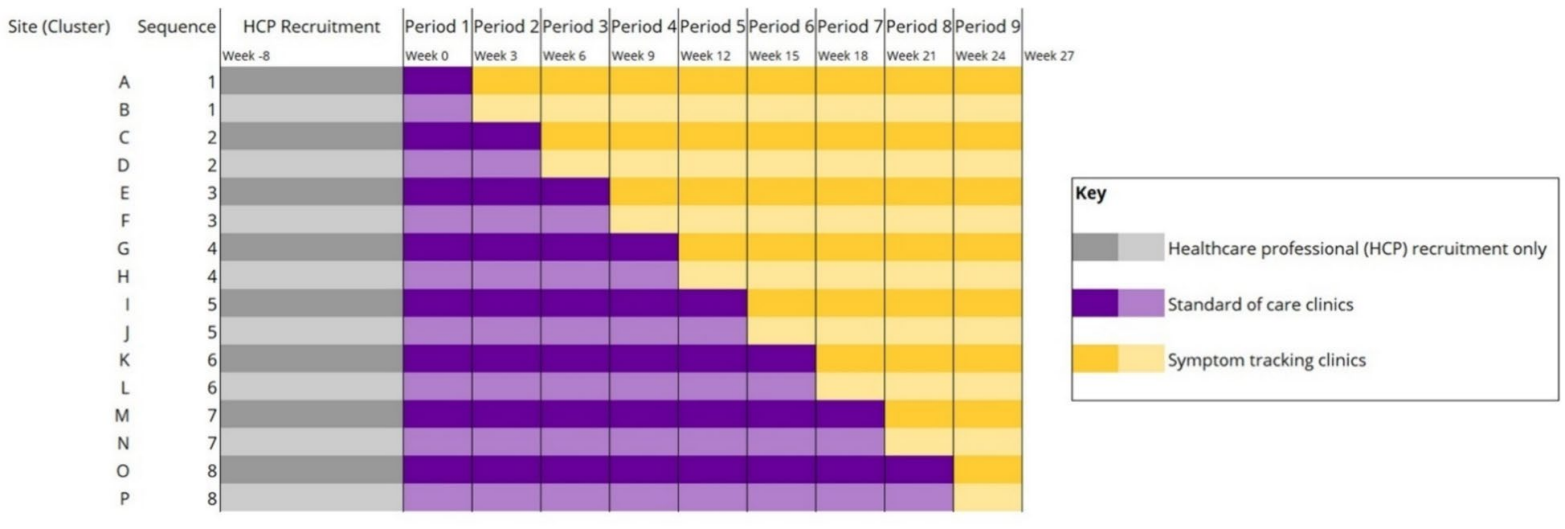
Stepped wedge cluster randomized controlled trial design. Healthcare professionals are primarily recruited during an 8-week run-in period (healthcare professional recruitment only; weeks − 8 to 0). All clusters recruit patients for a total of 27 weeks (6.2 months). All sequences initially recruit to clinic visits offering standard of care (control group). In pairs of two (i.e. sequences), clusters are randomly allocated to switch to clinic visits offering integrated symptom tracking (intervention group). All patient participants are followed up for 12 ± 3 months from the time of their allocation

This is a non-commercial, superiority, stepped wedge cluster randomized controlled trial with a continuous recruitment short exposure design [35]. Individual clusters (i.e. hospital sites) continually recruit patients throughout the trial, initially offering clinic visits according to standard of care and then switching to visits with integrated symptom tracking. Figure 1 shows how a sequence (i.e. pair of sites) switches over in eight steps (i.e. every 3-week period to offer integrated symptom tracking. The order in which sequences switch is randomized (see ‘ Assignment of intervention: allocation and blinding’).

Recruited patients are allocated to receiving either clinic visits with standard of care (i.e. control) or visits with integrated symptom tracking (i.e. intervention) depending on the period in which they are recruited; they continue to receive the clinic visit type they are initially allocated to, including after their cluster has switched over.

Patient participants have at least one baseline and one follow-up visit at 12 months. The final follow-up visit falls within 3 months either side of the 12-month follow-up date to allow for routine outpatient visit scheduling. The rheumatology teams may schedule additional clinic visits during the follow-up period in accordance with usual clinical practice; we will include the data from these additional visits in the analyses.

As part of a hybrid effectiveness-implementation study design [34], we gather information on implementation through the parallel process evaluation. For this, we invite patient participants, healthcare professionals, people involved in implementing integrated symptom tracking, and those eligible but declining trial participation to take part in interviews and/or observations.

### Study setting

This multi-centre trial involves 16 rheumatology outpatient departments (i.e. clusters) providing secondary care for people with RA; supplementary Table S1 contains a list of all participating hospital sites. Sites were eligible as a cluster if they were not yet using integrated symptom tracking as part of their standard of care but had staff who were willing to discuss symptom tracking data with patients as part of clinic visits as well as to collect trial outcome data. Staff members include healthcare professionals, such as rheumatology consultants, registrars and specialist nurses, who are directly responsible for patient care (i.e. conducting patient consultations, assessing disease activity, and/or determining and changing patient management and treatment). Depending on the cluster, these may also include other allied health professionals, such as consultant specialist physiotherapists or specialist pharmacists.

### Interventions

#### Intervention description

##### Integrated symptom tracking (i.e. intervention)

**Fig. 2:**
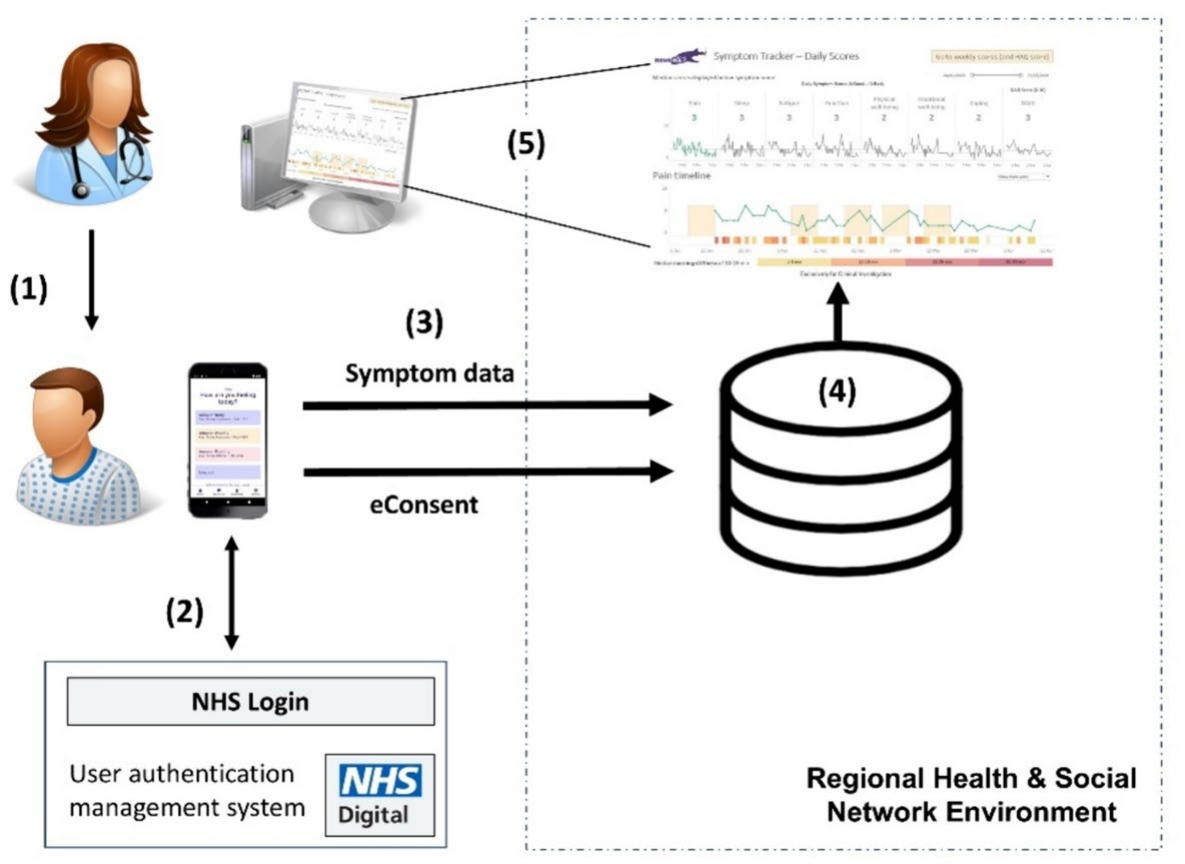
Overview of key element of the integrated symptom tracking intervention. (1) Upon allocation to the intervention group, a patient is ‘prescribed’ integrated symptom tracking through an app they can use on their own smartphone or other mobile device; (2) Using a national user authentication management system (NHS Login), patients securely authenticate themselves; (3) Once authenticated, patients can submit their daily, weekly and monthly data. Prior to the first submission, they are asked to provide electronic consent for reuse of their data for research; (4) Patients’ app data is stored in a regional data repository alongside other, already available clinical data; (5) Healthcare professionals view the app data via an interactive dashboard accessible in clinic within a patient-instance in their electronic health record system

Figure 2 gives an overview of the key elements of the intervention, which we have described in more detail below in line with the Template for Intervention Description and Replication (TIDierR) reporting guidance [36] (see Additional file 2 for completed checklist). Supplementary Figure S1 contains a logic model presenting our hypothesis of how the intervention works.

Patients allocated to the intervention group are ‘prescribed’ integrated symptom tracking, i.e. they receive instructions via email on how to set-up, register with, and use an app (i.e. the ‘REMORA app’) on their smartphone or other mobile device to track their symptoms and other patient-reported aspects of living with RA. The instructions also include how to set up or manage a Google account or Apple ID (for downloading the app from the respective app stores), as well as a National Health Service (NHS) login account (to facilitate secure user authentication; see [37]). As part of setting up, they are asked to electronically consent to their app data being used for research. The instruction and all other materials for patient participants are available in the ‘Information for participants’ section on the REMORA website [38].

Once set up, notifications within the REMORA app will prompt daily, weekly and monthly question sets to be completed during the 12 ± 3-month follow-up period; supplementary Table S2 describes the question sets in more detail. Patients receive notifications to remind them to submit their reports. Daily data can be submitted multiple times and at any time of day, with only the final value being viewable during clinic visits. Weekly and monthly questions can only be submitted when prompted. Within the app, patients can visualize and compare their symptom scores over time as graphs. An interactive version of the app is available online [39], with screenshots displayed in Figure S2. Patients have clinic visits in accordance with usual clinical practice and are informed that their tracked symptom data will only be reviewed during these visits and not in-between.

Patient data reported via the app is stored in a regional data repository, from where it is visualized in an interactive REMORA dashboard (see supplementary Fig S3 for screenshots). Consented healthcare professionals receive training [40] prior to trial start on how to use the dashboard for discerning patterns of time-varying disease activity since the patient’s last clinic visit. They can access it from within a patient-instance in their local electronic health record system without needing to log into a separate system. The dashboard consists of two main screens, one visualizing the daily app data, and one visualizing app data submitted weekly and monthly. Healthcare professionals can switch between these options and select any data item to be displayed as a graph for a customizable period. The dashboard is expected to augment rather than replace verbal questions and history taking as part of usual clinical practice but otherwise it is up to healthcare professionals’ discretion how they use the dashboard and discuss the symptom data during clinic visits.

##### Standard of care (i.e. control)

Patient participants allocated to the control group have clinic visits in accordance with usual clinical practice. During these visits, evaluation of how a patient has been since the last appointment is limited to the healthcare professional using verbal questions and history taking as per standard of care. No tracked symptom data is available for review and patients do not have access to the REMORA app. This enables us to evaluate whether the additional effort of implementing integrated symptom tracking into clinical systems and pathways provides benefit compared to hospital outpatient rheumatology services as currently delivered by NHS England.

##### Criteria for discontinuing or modifying allocated interventions

There are no special criteria for discontinuing or modifying allocated interventions. All participants may withdraw from the study at any time, and in addition to a participant formally withdrawing consent, the co-principal investigators can withdraw participants from the study in the event (a) a participant dies, (b) a participant’s capacity to consent changes, or (c) the trial’s sponsor or funder decide to terminate the study (e.g. in the unlikely event of a significant numbers of unanticipated serious safety events, or of sites withdrawing to the extent it invalidates the scientific credibility of the study).

Patient participants not using the intervention as intended are not automatically withdrawn and, unless they formally withdraw, continue to receive requests for data collection. Where a healthcare professional no longer wishes to participate, any consented patient participants under their care are withdrawn with guidance by the study team. If a healthcare professional withdraws because they relocate to another hospital, the colleague taking over their patient list will be approached for REMORA2 trial if not already participating. This will allow consented patients to continue on trial.

##### Relevant concomitant care permitted or prohibited during the trial

No restrictions or changes to medications or other treatments, or additional clinic visits are required as part of the REMORA2 trial. Patient participants continue to be followed up and use all medications as prescribed by their care team throughout the study.

##### Provisions for post-trial care

Once a patient participant has completed their final clinic visit in the 12 ± 3-month follow-up window, they are no longer considered to be on trial and are advised in writing that they have completed the study. Those allocated to the intervention group return to receiving standard of care only, and the REMORA app and interactive dashboard will be deactivated. There is no anticipated harm and compensation for trial participation.

#### Assignment of intervention: allocation and blinding

##### Sequence generation

As illustrated in Fig. 1, the 16 clusters are randomly assigned a site letter (A-P) by the programme manager independently of the trial statistician. Using computer-generated simple randomization, the trial statistician independently and randomly allocates letters A-P to eight sequences, numbered 1–8. The numeric order of the sequences reflects the order in which clusters switch over from standard of care to integrated symptom tracking, with sequence 1 switching over after the first 3-week period and sequence 8 at 24 weeks for the eighth and final step.

##### Concealment, implementation and blinding

The list of site names and letter allocations is stored in a password-protected file that only the programme manager has access to. The password-protected list of sequence numbers can only be accessed by the programme manager and trial statistician.

All sites will initially open to recruit to clinic visits offering standard of care. At the end of each 3-week period, the programme manager advises the study team which sequence is due to switch over to clinic visits offering integrated symptom tracking. The study team uses letters rather than site names to refer to clusters during meetings and in documentation to ensure the trial statistician remains blinded to the clusters’ identities. Similarly, the trial statistician is blinded to allocation of individual participants. Together, this reduces the risk of undue influence on the analyses and interpretation of results. The trial statistician remains blinded until the end of the REMORA2 trial when the primary and secondary outcome analyses have been completed. We do not foresee any circumstances or procedures that would require revealing a cluster’s or participant’s allocation to the trial statistician.

To reduce the risk of recruitment bias, clusters are not informed of their time of switch over from standard of care to integrated symptom tracking. This means that, until the first patient-reported symptom data appears in the interactive dashboard in the electronic health record, patients approached to participate cannot be informed by the site at the time of recruitment about whether their hospital is currently offering clinics with or without integrated symptom tracking. Where plausible, site team members who are not directly responsible for patient care but are involved in recruitment are kept unaware of the current intervention type being offered until the end of the recruitment period.

As it is not possible to blind patients from the intervention, they receive information about their allocation upon consenting to take part; allocation is managed by the study team. Patient-facing materials have been designed to present the control and intervention conditions in equipoise as much as possible to minimize influence on treatment beliefs. Similarly, it is not possible to blind healthcare professionals directly responsible for patient care due to patient-reported symptom data becoming available in the interactive dashboard once patient participants start tracking as part of the intervention.

#### Strategies to improve intervention uptake and adherence (i.e. intervention fidelity)

The REMORA2 trial employs several strategies to promote participants’ uptake of and adherence to the interventions. These have been informed by the findings from our feasibility trial [32], as well as by our patient and public involvement and engagement (PPIE) group. We describe the trial’s PPIE activities and outcomes to date below before providing further details on the promotional strategies.

##### Patient and public involvement and engagement (PPIE)

PPIE has been part of the REMORA programme ever since its inception in 2015. In the first phase, the group provided continuous advice and constructive criticism on the development and use of the initial version of the REMORA app [15]. The current REMORA2 trial is supported by a dedicated PPIE group consisting of nine members, all with lived experience of RA (see under *Our team* on the programme’s website [38]). They meet at least quarterly. KS and PAH are members who chair and co-chair the group, respectively.

Our PPIE approach aligns with the UK standards for public involvement in research [41]. This includes: inclusive opportunities (we expanded our original PPIE group to increase diversity and ensure members feel supported to provide input); working together (we have co-produced terms of reference and reimburse members for their time according to national guidance); learning and support (members receive training in line with their needs); governance (KS and PAH represent the PPIE group in the programme’s committees (see *Oversight and monitoring*) where they contribute to decision-making); communications (members co-produce patient information and dissemination materials); and impact (we continuously evaluate our PPIE activities and track impact on, e.g. digital inclusion).

The PPIE group informed the trial design by endorsing clinician-reported disease activity as the trial’s primary clinical outcome, while also collectively providing a clear steer that quality of life and shared decision making are the two most important secondary outcomes from a patient perspective. They collaborated with the study team to co-produce patient-facing materials and procedures with equality, inclusion and diversity of patient trial participation in mind. Examples of other key contributions from the PPIE work to date include as follows: creation of the ‘Information for participants’ section on the study’s website [38] to inform patient participants about aspects of the REMORA app and the trial; production of a joint self-assessment training video for patients [42]; and a PPIE-led helpdesk to handle patient participant queries.

##### Strategies for patient participants

Patient participants allocated to the intervention group will receive a mix of (up to five) e-mail and telephone call reminders about downloading, setting up and submitting their first symptom report using the REMORA app. Throughout the trial, they can request technical and other support via a telephone and email helpdesk during office hours; this includes peer support offered via the PPIE-led helpdesk.

Once patient participants have started using the app, they receive in-app reminders for submitting their daily/weekly/monthly reports. Patient participants who are not engaging with symptom tracking (defined as submitted < 50% of daily symptoms in the 14 days after tracking commenced, and monthly thereafter) receive (up to five) further email reminders.

##### Strategies for healthcare professionals

We provide physical reminders for healthcare professionals to review patient-reported symptom data using the interactive REMORA dashboard and collect the primary outcome data. This includes a Disease Activity Score for 28 joints (DAS28) desktop assessment tool, paper case report forms (CRFs) with REMORA2 logo, and REMORA pens and stickers. We also invite healthcare professionals to wear a REMORA-branded lanyard and pin badge to help patient participants mention their involvement in the study when they have a clinic visit (see Figure S4 for a photo of REMORA-branded materials). Where possible, clusters incorporate locally tailored reminders, such as flagging patients’ participation in outpatient letters or in electronic health records.

### Participants

#### Eligibility criteria

There are five participant groups: (1) Healthcare professionals participating in the trial, (2) Patients participating in the trial, (3) Eligible healthcare professionals who decline trial participation, (4) Eligible patients who decline trial participation and (5) Professionals or volunteers involved in implementing integrated symptom tracking. All should be willing and able to provide full informed consent. We summarize further eligibility criteria for each group below and in supplementary Table S3.

##### Healthcare professionals participating in the trial

Healthcare professionals are eligible to take part in the REMORA2 trial if they are (a) responsible for the assessment and treatment of people with RA, (b) have access to the hospital’s electronic health record system as part of routine care delivery and (c) are willing to review the interactive REMORA dashboard during clinical visits with consented patients in the intervention group.

Healthcare professionals taking part in the trial can also participate in an interview and/or clinic observations as part of the process evaluation if they are willing.

##### Patients participating in the trial

Adult patients (18 years or older) are eligible to take part in the REMORA2 trial if they (a) are under the care of a consented healthcare professional, (b) have active probable/definite RA (see Table S3 for definition), (c) have regular access to an app-compatible, internet-connected mobile device and a valid email address, (d) are able to follow the intervention set-up instructions independently or with support (see ‘ Intervention description’ for details), (e) have a record with clinical data in the regional data repository (see element 4 in Fig. 2) and (f) speak and understand English, or are supported by someone who does.

Patients taking part in the REMORA2 trial can also participate in an interview and/or clinic observation as part of the process evaluation, if they are willing and did not previously participate in the feasibility trial [32].

##### Eligible healthcare professionals who decline trial participation

Healthcare professionals who are a member of the care team in one of the clusters and are eligible but decline to take part in the trial, are eligible for an interview as part of the process evaluation if they are willing.

##### Eligible patients who decline trial participation

Patients who are eligible but declined to participate in the REMORA2 trial are eligible for an interview as part of the process evaluation, if they are willing, regardless of whether they previously took part in the feasibility trial [32].

##### Professionals or volunteers involved in the implementation of integrated symptom tracking (implementers)

Healthcare professionals, other professionals (e.g. IT staff, information governance experts), or volunteers are eligible for taking part in an interview for the process evaluation if they have been involved in the technical and/or organizational implementation of the REMORA2 intervention (i.e. integrated symptom tracking) at a local (e.g. hospital), regional (e.g. data repository) or national (e.g. NHS login) level. We will refer to this participant group as ‘implementers’ in the remainder of the manuscript.

#### Recruitment

##### Healthcare professionals

Trained and delegated members from teams within clusters are responsible for recruitment, supported by the study team. Healthcare professionals are primarily recruited prior to patient recruitment commencing (see Fig. 1), with further recruitment taking place throughout the REMORA2 trial as needed (e.g. due to staff changes). All eligible healthcare professionals receive training from the study team on how to use the interactive REMORA dashboard (see ‘ Intervention description’), as well as a participant information sheet with adequate time to consider if they wish to participate, and the opportunity to ask further questions.

Those declining trial participation who provided consent to be contacted (see ‘ Informed consent’) receive an invitation for the process evaluation by taking part in an interview, covered in a separate participant information sheet.

##### Patients

Potentially eligible patients are primarily identified via case note review by trained and delegated members from teams within clusters. Localized recruitment posters displayed in patient areas advertise the REMORA2 trial to people who have not yet been approached, and healthcare professionals have localized REMORA ‘business cards’ they can give to patients who may be eligible and interested. The posters and cards include a URL and QR code leading to the study website [38], as well as details for whom to contact for expressing an interest or for more information.

Following identification, cluster team members approach patients in-person in clinic, by telephone, or via mail to discuss the trial and confirm eligibility via a brief screening procedure. Interested individuals receive a participant information sheet (available on [40]). with adequate time to consider if they wish to participate, and the opportunity to ask further questions. To ensure adequate patient participant enrolment, all cluster teams receive weekly calls from the study team to discuss and address any recruitment barriers, monthly newsletters with updated cluster-level recruitment numbers compared to targets, and site visits from the programme manager to identify further recruitment opportunities as required.

Patients declining trial participation who provided consent to be contacted (see ‘ Informed consent’) receive an invitation for the process evaluation by taking part in an interview, covered in a separate participant information sheet.

##### Implementers

As part of the process evaluation, people who have been involved in implementing the intervention are identified by the programme manager and other study team members, as well as by cluster contacts. The study team contact those eligible and provide them with a participant information sheet, allowing adequate time to consider participation and giving people the opportunity to ask questions.

#### Informed consent

##### Who will take informed consent?

Trained and delegated members from teams within clusters or from the study team consent patients and healthcare professionals to participate in the trial or to be contacted for an interview (e.g. if declining trial participation). Completed consent forms are uploaded electronically into a ‘participant record’ on the REMORA2 trial database hosted on REDCap and managed by the study team. A model consent form is publicly available in our trial repository [40].

##### Additional consent provisions for collection and use of participant data and biological specimens

All participants are asked to consent for reuse of their data for future research. Reuse of research data will be at the discretion of the co-principal investigators and will only be granted where adequate data protection measures are in place that ensure data confidentiality. We are not collecting biological specimens in this trial.

#### Participant timeline

**Fig. 3:**
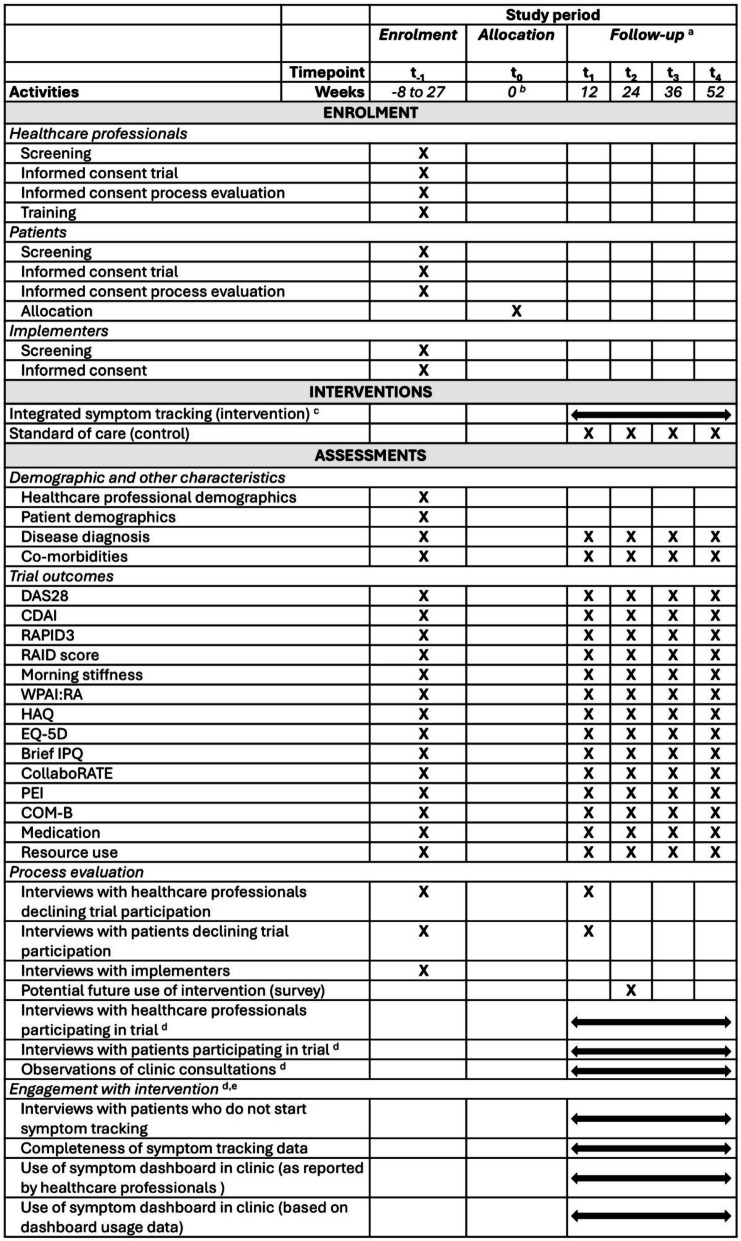
SPIRIT diagram of schedule of activities. Abbreviations: Brief IPQ: Brief Illness Perception Questionnaire; CDAI: Clinical Disease Activity Index; CollaboRATE: share decision making questionnaire; COM-B: Capabilities, Opportunities, Motivations for Behaviour change questionnaire; DAS28: Disease Activity Score for 28 joints; EQ-5D-5L: EuroQol five-dimension scale questionnaire; HAQ: Health Assessment Questionnaire; PEI: Patient Enablement Instrument; RAID: Rheumatoid Arthritis Impact of Disease score; RAPID-3: Routine Assessment of Patient Index Data 3; WPAI-RA: Work Productivity and Activity Impairment Questionnaire: Rheumatoid Arthritis. **a** Frequency and time points of follow-up appointments are scheduled by the rheumatology team and according to clinical need. No additional visits are mandated as part of the REMORA2 trial. Procedures listed reflect the maximum number of expected activities; **b** Clinical data extracted from the electronic health record at t_0_ comprises data collected at the most recent appointment within the period from 6 months before or 1 month after the time of allocation; **c** For patients who have a clinic visit between months 9 and 12, the REMORA app is deactivated after 12 months. Those who do not have a clinic visit in this period are asked to continue tracking their symptoms beyond 12 months, with the REMORA app being deactivated as soon as they had their final clinic visit; **d** Assessments take place throughout the follow-up phase with participants only being interviewed/observed once; **e** Intervention group only

Figure 3 shows the time schedule of enrolment, interventions and assessments. Implementation of the intervention (e.g. technical integration of the interactive dashboard into local electronic health record systems, staff training on how to use the dashboard) is completed prior to recruitment commencing, so no further implementation (or washout) time is required at the point of clusters switching over.

### Outcomes

#### Primary outcome

As shown in Table 1, the primary outcome (to address objective 1) is a difference in the marginal mean disease activity score (DAS) as measured on the DAS28 with C-reactive protein (DAS28-CRP), at 12 ± 3 months follow-up between the standard of care and the integrated symptom tracking groups, after accounting for covariates (including baseline DAS28-CRP, study site, follow-up length and others). The DAS28 is a composite disease activity score that incorporates information about swollen joints, tender joints, acute phase response and global assessment [43]. It is used widely in clinical practice and clinical trials and forms the foundation of internationally accepted treatment response criteria [44]. The DAS28-CRP is a subset of the DAS28, which uses the CRP blood test result to derive the composite score. We opted for CRP rather than erythrocyte sedimentation rate (an alternative blood test for calculating DAS28) based on what was most used by participating hospital sites.

**Table 1:** Outcomes per objective, including measurement variables, data collection methods, and analysis metrics

Objective	Outcome	Measurement variable	Data collection method	Analysis metric
**Primary objective**				
1	Clinician-reported disease activity	DAS28-CRP [43]^a^	EHR extraction	Difference in marginal mean DAS28-CRP score at t4 between control and intervention group^b^
**Secondary objectives**				
2a	Treatment response categories	EULAR response categories [44]	Derived from DAS28-CRP	Difference in proportion of participants meeting good/moderate/non-response at t4 between control and intervention group^b^
	Patient-reported disease activity	RAPID-3 [45]	Patient web survey	Difference in RAPID-3 total score at t4 between control and intervention group^b^
		Patient global assessment	Patient web survey	Difference in patient global assessment score at t4 between control and intervention group ^b^
		RAID [46]	Patient web survey	Difference in RAID score at t4 between control and intervention group^b^
		CDAI [47]^c^	Patient web survey	Difference in CDAI score at t4 between control and intervention group^b^
	Joint involvement and severity	TJC28	EHR extraction	Difference in TJC28 score at t4 between control and intervention group^b^
		SJC28	EHR extraction	Difference in SJC28 score at t4 between control and intervention group^b^
	Medication use	n/a	EHR extraction	Difference in medication use of prescribed medication at t4 between control and intervention group^b, d^
	Work impairment	WPAI-RA [48, 49]	Patient web survey	Difference in WPAI-RA score at t4 between control and intervention group^b^
	Quality of life	EQ-5D-5L [50]	Patient web survey	Difference in EQ-5D-5L health profiles, index score and quality-adjusted life years at t4 between control and intervention group^b^
	Disability	HAQ [51]	Patient web survey	Difference in HAQ score at t4 between control and intervention group^b^
2b	Patients’ experience of shared decision-making during clinic visits	CollaboRATE [52]	Patient web survey	Difference in CollaboRATE score at t4 between control and intervention group^b^
	Researcher-observed shared decision-making processes during clinic visits	OPTION5 [53]	Clinic observations	Difference in OPTION score at t4 between control and intervention group^b^
	Patients’ experience of clinic visit quality	PEI [54]	Patient web survey	Difference in PEI score at t4 between control and intervention group^b^
	Patients’ self-management	COM-B [55]	Patient web survey	Difference in COM-B score at t4 between control and intervention group^b^
	Patients’ understanding of their RA	Brief IPQ [56]	Patient web survey	Difference in brief IPQ score at t4 between control and intervention group^b^
	Perceptions of self-management, clinic visits, and decision-making processes	n/a	Interviews with patients and healthcare professionals participating in trial; clinic observations	Comparative thematic and conversation analysis of transcripts between control and intervention group
3	Expectations, experiences and views on the acceptability and usefulness of integrated symptom tracking	n/a	Interviews with patients and healthcare professionals participating in trial or declining participation; interviews with implementers; clinic observations; CRFs; patient web survey	Thematic and conversation analysis of transcripts; descriptive analysis of survey responses and CRFs
	Facilitators and barriers to behaviour change and intervention implementation and uptake	n/a		
4	Quality of life	EQ-5D-5L [50]	Patient web survey	Difference in EQ-5D-5L health profiles, index score and quality-adjusted life years at t4 between control and intervention group^b^
	Duration of consultation	n/a	Clinic observations	Difference in cumulative consultation duration at t4 between control and intervention group^b^
	Medication use	n/a	EHR extraction	Difference in medication use of prescribed medication at t4 between control and intervention group^b, d^
	Resource use^e^	n/a	EHR extraction; patient web survey	Difference in cumulative resource use duration at t4 between control and intervention group^b^
	Patient travel time and costs^f^	n/a	Patient web survey	Difference in cumulative travel time and costs at t4 between control and intervention group^b^
	Healthcare professionals’ engagement with integrated symptom tracking	n/a	Interactive dashboard usage data; CRFs	Descriptive analysis of number and duration of views of interactive dashboard
	Patients’ engagement with integrated symptom tracking	n/a	Symptom completion data	Descriptive analysis of number of completed daily, weekly and monthly questionnaires
5	All outcomes as listed above for objectives 1–4	n/a	n/a	Exploratory analyses of relationships between primary and secondary indicators of disease activity (objectives 1 and 2a) and other secondary outcomes

*CDAI* Clinical Disease Activity Index for RA, *CollaboRATE* share decision making questionnaire, *COM-B* Capabilities, Opportunities, Motivations for Behaviour change questionnaire, *CRF* Case report form, *DAS28-CRP* Disease Activity Score for 28 joints using C-reactive protein, *EHR* Electronic health record, *EULAR* European Alliance of Associations for Rheumatology, *EQ-5D-5L* EuroQol five-dimension scale questionnaire, *HAQ* Health Assessment Questionnaire, *IPQ* Illness Perception Questionnaire, *PEI* Patient Enablement Instrument, *RA* rheumatoid arthritis, *RAID* Rheumatoid Arthritis Impact of Disease score, *RAPID-3* Routine Assessment of Patient Index Data 3, *SJC28* Swollen Joint Count for 28 joints, *TJC28* Tender Joint Count for 28 joints, *WPAI-RA* Work Productivity and Activity Impairment questionnaire: Rheumatoid Arthritis

^a^For remote consultations, we collected a CDAI score [47] instead of a DAS28 as calculation of the former does not require a blood test

^b^After accounting for measured variables that improve estimate precision, including cluster and outcome assessment at t0 (i.e. baseline); t4 is the time of final follow-up at 12 ± 3 months

^c^Our PPIE group developed a ‘Checking your tender and swollen joints’ section on the study’s website [38] with instructions and a training video for patients on how to self-assess and count tender and swollen joints

^d^Medication use at baseline includes past (i.e. previous 6 months) and current exposures, with any changes to RA medication recorded at all subsequent follow-up visits

^e^Includes number and dates of in-person rheumatology consultations and related travel time/costs, telephone rheumatology consultations, general practitioner consultations and hospital admissions

^f^Related to in-person rheumatology outpatients visits

#### Secondary outcomes

Table 1 lists all secondary outcomes for addressing objectives 2–5, including their specific measurement variables, analysis metric/approach and, where appropriate, the method of aggregation; no continuous outcomes will be analysed as categorical. Time points for each outcome can be found in Fig. 2.

##### Sample size

The accepted minimal clinically important difference for effect of a direct novel disease modifying therapy for RA is a change of 1.2 points on the DAS28 [57]. As our intervention is modifying disease indirectly, we are anticipating a smaller target difference of 0.6 points in the DAS28, with an expected standard deviation of 1.2 [57].

In the absence of established methodology for sample size calculations for trials with a continuous recruitment short exposure design, we used the ShinyCRT calculator [58, 59] with the cross-sectional design setting to produce a conservative (i.e. greater) sample size estimate. We estimated that we needed to include 432 patient participants in the analysis (i.e. 27 participants per cluster) to achieve at least 90% power for detecting the target difference under the following assumptions: a discrete time decay within-cluster correlation structure; varying cluster sizes with a coefficient of variation of cluster size of 0.15 (based on accruing roughly 27 ± 4 patient participants per cluster); a between-groups a type-I error rate (alpha) of 0.05; an intra-class correlation (ICC) of 0.05; a cluster auto-correlation of 0.8; and 16 clusters switching over in pairs of two across eight steps. In the absence of available shared datasets and published trials relevant to ours, we formulated our assumptions regarding the ICC and cluster auto-correlation in consultation with the clinical trial design expert on the REMORA2 programme steering committee (see under ‘ Oversight and monitoring’ below), who advised on commonly used and relatively conservative figures that would give us ample sample size in a range of potential scenarios.

Based on findings from the feasibility trial [32], we anticipate 30% of recruited patient participants allocated to the intervention group may not initiate symptom tracking, with a further 15% across both groups being lost to follow-up due to drop-out/withdrawal. To compensate for this attrition and achieve the required analysis sample size of 432, we aim to recruit a total of 736 participants (i.e. 46 per site).

There are no formal interim analyses planned, thus we do not anticipate any adjustments to the sample size.

### Data collection and management

#### Plans for assessment and collection of outcome and other data

Table 1 lists the data collection methods and study instruments for all outcomes, with Fig. 2 showing when assessments take place. The sections below further describe the data collection methods.

##### Electronic questionnaire for healthcare professional demographics

After completing the training and providing informed consent, healthcare professionals participating in the trial receive an invitation via email to complete an online questionnaire on their demographics (age, gender, ethnicity, role, years of rheumatology experience).

##### Electronic health record data extraction

Delegated staff at clusters use an eCRF to enter data extracted from the electronic health record system on clinician-reported disease activity, joint involvement and severity and medication and resource use. For the baseline assessment, this additionally includes patient demographics (gender, date of birth, ethnicity, smoking status, body mass index) and comorbidities.

The baseline assessment is based on information recorded for the clinic visit on, or closest to, the date of recruitment, but within a time window of 6 months prior to or 1 month after consent. Follow-up assessments are performed at each clinic visit during the 12 ± 3-month follow-up window but since these visits are scheduled by the care team as required, no pre-defined schedule of follow-up data extractions has been set.

Healthcare professionals complete paper versions of the eCRFs during follow-up visits to aid data collection.

##### Patient web survey

We use an electronic questionnaire to collect patient-reported data from patient participants on disease activity, joint involvement and severity, work impairment, quality of life, disability, experience of care, self-management, understanding of their RA, resource use and travel time and costs related to in-person rheumatology outpatients visits. The baseline assessment additionally includes patient demographics (year of birth, gender, ethnicity, smoking status, postcode (to derive level of relative socio-economic deprivation), income, educational attainment).

For the baseline assessment, all patient trial participants receive instructions for completing the questionnaire at the time of allocation (irrespective of allocation group). Follow-up assessments take place at 3, 6, 9 and 12 months post-allocation.

There is an additional online questionnaire at 6 months asking patient participants to share their thoughts on the benefits of a range of potential future developments/uses of integrated symptom tracking.

##### Paper case report forms

In addition to duplicating the items on the eCRF, the abovementioned paper CRF also includes follow-up assessments of healthcare professionals’ use of the interactive REMORA dashboard (if and at what time they viewed the tracked symptom data (e.g. before or during the clinic visit), and if and how it was useful/informed the consultation).

##### Interactive dashboard usage data (intervention group only)

We collect data on the date, time and duration of a user session for each occasion the interactive REMORA dashboard is opened. This data is supplied by the two providers of the regional data repositories (see element 4 in Fig. 2) in Greater Manchester and Northwest London and is extracted from their systems’ usage logs.

##### Symptom completion data (intervention group only)

Similar to previous phases of REMORA [15, 32], completion data for the daily, weekly and monthly questionnaires comes from the app data and is provided by the software team hosting and supporting the REMORA app.

##### Clinic observations

A member of the study team conducts in-person, non-participant clinic observations using an observation template that covers the length and process of the consultation, as well as completion of the OPTION5 tool to assess to what extent shared decision-making is happening [53]. For clinic visits with patient participants in the intervention group, we also explore if, how and when the patient-reported symptom data are reviewed and discussed. Visits are audio-recorded if patient and healthcare professional have consented to this; else, the observer takes notes. Audio-recordings are transcribed verbatim by a professional secretarial service.

##### Interviews

All interviews are individual and semi-structured. A member of the study team conducts interviews in-person or via telephone or video conferencing, depending on participant preference. Interviews are expected to last between 30 min and 1 h and cover a range of topics, depending on the participant type (see supplementary Table S4 for further details on interview topics). Where possible, we use purposive sampling to maximize variation of interviewees’ characteristics (e.g. age, gender, ethnicity, intervention, engagement level). The interviewer audio-records the interview where consent has been given or else takes notes. Audio-recordings are transcribed verbatim by a professional secretarial service.

#### Plans to promote participant retention and complete follow-up

Most strategies used to promote participant retention and complete follow-up are outlined under ‘Strategies to improve intervention uptake and adherence (i.e. intervention fidelity)’*.* In addition, patient participants receive a combination of up to three email and text reminders to complete each patient web survey. Patients currently on trial receive quarterly patient newsletters, with content developed by our PPIE group.

#### Data management

##### Data collected at site

As described above, clusters collect data using paper and electronic CRFs. All eCRFs are hosted and stored on REDCap, a secure, web-based platform designed to support research data capture. Paper CRFs are transcribed into electronic format by delegated cluster staff and stored on site. The cluster’s local principal investigator is responsible for ensuring the accuracy, completeness, legibility and timely provision of the data reported in the (e)CRFs.

##### Data collected directly from patient participants

The online survey for collecting patient-reported data is hosted on REDCap, with some of the questionnaire items also being collected via the REMORA smartphone app by patient participants allocated to integrated symptom tracking.

##### Data collected through interviews and observations

Where consent has been given, all interviews and observed clinics are audio-recorded using an encrypted device and transferred to secure servers at the earliest opportunity. Study team members may create written field notes on paper (e.g. if consent to recording is not offered, or to provide additional context to discussions). Paper notes are scanned, stored on the secure servers and then destroyed as soon as practically possible.

#### Confidentiality

We regard all personal data collected as part the REMORA2 trial as confidential, and no information allowing (direct or indirect) identification of individuals is released into the public domain. One measure to ensure confidentiality is the REMORAid. This is a unique participant identifier (or trial number) we assign to all participants, comprising a two-letter site code and a randomly generated five-digit number. Patient participants in the integrated symptom tracking group use the REMORAid to set up the REMORA app prior to submitting their first symptom report, while the study team use it to internally link participant-level data throughout the trial.

Furthermore, the programme manager, delegated cluster staff and the study team maintain all essential documents in a way that facilitates management, audit and inspection of the study. Essential documents are documents that individually and collectively permit evaluation of the conduct of the study and substantiate the quality of the collected data. Any essential documents required to be held by the co-principal investigators are stored by the study team so that these are readily available, upon request, to the regulatory agency and sponsor for the minimum period as required by the regulator and UK Law. Medical files of patient participants are retained in accordance with national legislation.

All essential documents are securely stored, with access restricted to authorized personnel. Direct access is granted to restricted members of the study team involved in data management and analysis, as well as appropriate IT and governance staff at the sponsor institution.

#### Plans for collection, laboratory evaluation and storage of biological specimens for genetic or molecular analysis in this trial/future use

No biological specimens are collected for analysis in this trial or for future use. We only obtain laboratory values of inflammatory markers obtained at hospital sites as part of usual care.

### Statistical methods

#### Statistical methods for primary and secondary outcomes

This section summarizes the planned statistical methods for the primary and secondary quantitative outcomes listed in Table 1. For all analyses described below, we will take an intention-to-treat approach, i.e. we will include participants in the treatment group they are assigned to regardless of the extent to which they take up or adhere to the intervention. Further details, including on methods for qualitative outcomes, will be provided in the full statistical analysis plan, which we will draft with input from the trial’s programme steering committee and publish before we lock the trial data set.

We will report the trial results in accordance with the CONSORT stepped wedge trial extension [60].

##### Primary outcome analysis

The primary analysis will use a generalized linear mixed-effects regression model, with DAS28-CRP at 12 ± 3-months follow-up as the outcome [61, 62]. Model predictors include DAS28-CRP at baseline, follow-up length (in days) and intervention (standard of care or integrated symptom tracking), complemented with further relevant prognostic factors. We will include cluster as a random effect and report the ICC of the linear mixed effects models alongside model coefficients. We will report effect estimates as 95% confidence intervals and consider intervals excluding a null effect as statistically significant. As there is only a single primary outcome of interest, we will not adjust at the type-I error level.

Since our continuous outcome short exposure design means that each participant only receives one intervention, we are not expecting any carry-over effects.

##### Secondary outcome analyses

We will analyse continuous secondary outcomes measured at baseline and follow-up for both groups in a similar way as the primary outcome. For categorical secondary outcomes (e.g. EULAR response categories, medication use), we will use mixed-effects logistic models. Since we wish to estimate the conditional, rather than the marginal, odds ratio of the outcomes, we will not include baseline values as model predictors for these models [63]. We will report effect estimates as 95% confidence intervals and consider intervals excluding a null effect as statistically significant. Results for the secondary outcomes will be reported both as estimates without adjustment to the type-I error level, as well as with Ryan–Holm step-down Bonferroni procedure-adjusted 95% confidence intervals [64] to account for multiplicity given the number of secondary outcomes.

For objective 4 (cost-effectiveness), we will undertake a health economic evaluation from a health system perspective with a sensitivity analysis from a societal perspective. For the health system perspective, we will take unit costs from relevant publicly available sources, such as the Unit Costs for Health and Social Care [65], National Cost Collection Data for NHS England [66] and the British National Formulary [67]. The societal perspective sensitivity analysis will include broader costs outside of the healthcare system, including work productivity and travel time as reported by patient trial participants. We will calculate the incremental cost per quality-adjusted life-year gained by multiplying resource use by appropriate unit costs, and utilities calculated from the EQ-5D-5L. We will develop a generalized linear mixed-effects regression model to estimate mean values and 95% percentiles using non-parametric bootstrapping.

#### Methods for interim and additional analyses

Since we are expecting engagement with integrated symptom tracking to vary between patient participants in the integrated symptom tracking group, we will conduct and report sensitivity analyses for ‘best-case’ and ‘worst-case’ scenarios, i.e. assuming very high and very low engagement across participants, respectively.

There are no formal interim or subgroup analyses planned.

#### Methods in analysis to handle protocol non-adherence and any statistical methods to handle missing data

We have developed processes and materials to minimize non-adherence and missing data (see ‘Strategies to improve intervention uptake and adherence (i.e. intervention fidelity)’). Where missing data exists, we will summarize and report missingness in terms of outcome, data source (e.g. eCRF, patient web survey) and reasons for missingness (e.g. participant loss-to-follow-up, withdrawal, protocol deviation).

For the primary outcome analysis, we will use multiple imputation by chained equations where data points are assumed to be missing at random. We will detail multiple imputation model specifications in the full statistical analysis plan, but in general, we will seek to impute across all variables included in the primary and secondary analyses; particularly since we expect the primary outcome (DAS28-CRP) to correlate strongly with other, secondary measures of disease severity (e.g. clinical disease activity index [47]).

A sensitivity analysis will replicate the primary outcome analysis using complete case analysis (i.e. using only observed data from the non-imputed final dataset) and we will present its findings alongside those from the primary outcome analysis. Should the study team feel that data may not be missing not at random (e.g. because of unanticipated events), we will revise the statistical analysis plan to include additional analysis strategies for quantifying the robustness of the point estimate generated from the primary analysis model. Any additional sensitivity analyses will be presented in the main REMORA2 trial report.

#### Plans to give access to the full protocol, participant-level data and statistical code

We intent to make the full (‘operational’) REMORA2 trial protocol, statistical analysis code, statistical analysis plan and synthetic de-identified versions of the trial analysis dataset(s) publicly available in our trial repository [40]. Access to participant-level data for future, ancillary studies is only permissible on request to the trial’s co-principal investigators (SNVDV and WGD), and will be considered on a case-by-case basis (also see ‘Availability of data and materials’).

#### Analytical software

We are planning to undertake all quantitative analyses using the most recently validated version of R [68] or another appropriate validated statistical software package. We will stipulate the software and version in the relevant reports and publications. Code for analyses will be shared in our repository [40].

### Oversight and monitoring

#### Composition of the coordinating centre and trial steering committee

The coordinating centre is at the University of Manchester, where most of the study team is also based. There are three internal oversight groups. First, the REMORA2 programme management group comprises individuals responsible for the day-to-day management of the trial, as well as at least one member of the PPIE group. They meet monthly to monitor management of the programme, progress across workstreams, communications, trial outputs and future plans. Second, the REMORA2 trial management group, a subgroup of the programme management group, meets monthly to monitor all aspects of the conduct and progress of the trial, ensure protocol adherence and take appropriate action to ensure the quality of the research. Lastly, the scientific committee includes all study team members responsible for the scientific integrity of the trial, complemented with a PPIE group representative. This committee meets monthly to discuss methodological challenges (e.g. with data analysis), review preliminary findings, present recent advances in the field of integrated symptom tracking and provide oversight of publications in draft.

Furthermore, we have established the REMORA2 programme steering committee. It has external representation from an independent chairperson, one other independent expert, an independent statistician with stepped wedge trial expertise and one independent patient representative. It additionally has internal representation from the co-principal investigators and the programme manager. Other invitees may include a PPIE group member, study team members, a sponsor representative, or funder representatives, as needed. The committee meets at least twice annually.

Lastly, we have convened an independent expert group that meets once every 9–12 months to advise on future implementation and scalability of integrated symptom tracking beyond the REMORA2 trial. Membership includes representation of patient and professional organizations, commissioners, healthcare providers, digital health industry, academia and guideline and regulatory bodies.

#### Composition of the data monitoring committee, its role and reporting structure

The programme steering committee did not deem an independent data monitoring committee necessary for the REMORA2 trial. Instead, the committee has taken on this responsibility to review accruing data and monitor for reasons that may require trial discontinuation. As no interim analyses are planned, reasons for discontinuation will be unrelated to the primary analyses but may instead concern unlikely events, such as significant numbers of unanticipated serious safety events, or a high number of sites withdrawing.

#### Adverse event reporting and harms

The programme manager is notified (via a standard report form) by delegated cluster staff in case of any serious adverse events or any device deficiency that might have led to a serious adverse event if (a) suitable action has not been taken, or (b) intervention has not been made, or (c) circumstances have been less fortunate. We will follow-up all reported events to a satisfactory conclusion.

#### Frequency and plans for auditing trial conduct

We undertake on-site monitoring in line with our sponsor’s auditing requirements [69] using a risk-based strategy for data quality to ascertain the frequency and intensity of monitoring visits required. We will conduct additional monitoring as and when needed.

#### Plans for communicating important protocol amendments to relevant parties

The co-principal investigators review and approve any changes in research plans and activities. Where necessary, changes are submitted in writing to the research ethics committee, Medicines and Healthcare Products Regulatory Agency (MHRA) and local research and development teams at hospital sites for approval prior to enrolment into an amended protocol. The programme manager tracks the amendment history and coordinates dissemination to all participating hospital sites, members of the study team at the coordinating centre and other co-investigators. Amendments potentially impacting people’s appreciation of trial risks will also be shared with participants, alongside updating their informed consent if required.

### Dissemination plans

Upon completion of the study, members of the study team will analyse the data and produce and publish at least one final study report in a peer-reviewed journal; we do not intend to use professional writers. Further publications and conference abstracts will be published in consultation with the REMORA2 scientific committee and following authorization by the co-principal investigators, who will apply the International Committee of Medical Journal Authors guidance to confirm authorship eligibility. We inform participants of study findings via the regular newsletters.

## Discussion

Despite the ongoing digital transformation of healthcare services worldwide, the evidence for effectiveness and value for money to support the roll-out of digital health interventions remains scarce [70]. At the same time, the UK’s National Institute of Care Excellence evidence standards framework for digital health and care technologies [71] requires the highest-level evidence for active monitoring interventions, such as integrated symptom tracking, to be adopted into the NHS. The REMORA2 stepped wedge cluster randomized controlled trial addresses this by generating robust evidence about the comparative effectiveness and cost-effectiveness of integrated symptom tracking compared to standard of care in people living with RA. In addition, we are undertaking several parallel activities to prepare uptake of integrated symptom tracking beyond the trial. These include exploring influencing factors for behaviour change and implementation through the process evaluation; working with relevant regional and national stakeholders to ensure we learn transferrable lessons on integrating daily symptom tracking into the NHS to inform regional and national policy; and seeking regulatory approval of our integrated symptom tracking intervention as a medical device. Although the pathway for commissioning digital services in the NHS remains largely opaque, we hope that—by producing the required evidence coupled with the knowledge, materials and stakeholder support to facilitate implementation—we optimize the chances of integrated symptom tracking being adopted more widely if demonstrated (cost-)effective at the end of the trial.

Another parallel activity is the development of a governance framework and eConsent prototype. The aim is to enable future patients to provide consent from home for their symptom or other patient-generated health data to be used for the purpose of direct care, as well as for population health research. This acknowledges the added value of high-frequency, longitudinal patient-generated health data for addressing research questions that are important to patients but currently remain unanswered, such as ‘Which of these treatments is likely to work faster for me?’. Combining routinely collected electronic health record data on medication use with daily patient-reported symptoms, as we are doing in the REMORA2 trial, will create a powerful and unique dataset for comparative effectiveness research.

Integrated symptom tracking offers additional opportunities to change and improve healthcare services beyond those being evaluated within this trial. Although at present we intentionally restricted the scope to RA patients with active disease and to healthcare professionals only viewing the tracked symptom data during planned outpatient visits, others have started investigating using patient-generated symptom data to reduce clinic visit frequency for patients who are stable or in remission [26, 29, 31]. Integrated symptom tracking might also allow patients to be seen at times of need, or to receive ‘just in time’ supportive interventions, such as self-management advice when flaring. We hope that providing evidence for the benefits and cost-effectiveness of integrated symptom tracking at *planned* clinic visits will pave the way for scaling up this initial version into the NHS, thereby enabling further research and innovation for advancing services for people with long-term conditions to receive the best possible care at the right time.

## Trial status

Protocol Version number: Version 2.0 (5-Jul-2024).

Date patient participant recruitment began: 10-Jul-2024.

Expected date of recruitment completion: 15-Dec-2024.

## Supplementary Information

Additional file 1. Completed SPIRIT checklist.

Additional file 2. Completed TIDieR checklist.

Additional file 3. Supplementary materials.

## Data Availability

The data collected for the REMORA2 trial is detailed health data. Given the detailed nature of the dataset, and in-line with participants’ understanding of the proposed use of their data in the participant information sheet, access for research purposes is only permissible on request to the trial’s co-principal investigators (SNVDV and WGD). Requests to access the REMORA2 dataset will be considered on a case-by-case basis. For each data access request, we will assess the proposed use to ensure it is in-line with participants’ reasonable expectations and will apply the principals of data minimization and data confidentiality.

## Abbreviations

CRF: Case report form
CRP: C-reactive protein
DAS28: Disease activity score for 28 joints
ICC: Intra-class correlation
MHRA: Medicines and Healthcare Products Regulatory Agency
NHS: National health service
PPIE: Patient and public involvement and engagement
RA: Rheumatoid arthritis
REMORA: REmote MOnitoring of Rheumatoid Arthritis
SPIRIT: Standard Protocol Items: Recommendations for Interventional Trials
TIDieR: Template for Intervention Description and Replication
