# Supplementary material for: Remote monitoring of rheumatoid arthritis (REMORA): study protocol for a stepped wedge cluster randomized trial and process evaluation of an integrated symptom tracking intervention
Source: Trials. 2024 Oct 15;25:683. doi: 10.1186/s13063-024-08497-9 (PMC11481815; doi:10.1186/s13063-024-08497-9)
Supplement: Supplementary file 1 — Additional file 1. Completed SPIRIT checklist. [file 13063_2024_8497_MOESM1_ESM.pdf]

Additional file: Completed SPIRIT-Outcomes 2022 extension checklist

Section	Item No.	SPIRIT 2013 Item	SPIRIT-Outcomes 2022 item	Location Reported
**Administrative information**				
Title	1	Descriptive title identifying the study design, population, interventions, and, if applicable, trial acronym	-	Pg 1
Trial registration	2a	Trial identifier and registry name. If not yet registered, name of intended registry	-	Pg 4 (abstract), 5
	2b	All items from the World Health Organization Trial Registration Data Set	-	In ISRCTN trial registration record; throughout manuscript
Protocol version	3	Date and version identifier	-	Pg 5, 35
Funding	4	Sources and types of financial, material, and other support	-	Pg 5, 36
Roles and responsibilities	5a	Names, affiliations, and roles of protocol contributors	-	Pg 1, 35-36
	5b	Name and contact information for the trial sponsor	-	Pg 5
	5c	Role of study sponsor and funders, if any, in study design; collection, management, analysis, and interpretation of data; writing of the report; and the decision to submit the report for publication, including whether they will have ultimate authority over any of these activities	-	Pg 5
	5d	Composition, roles, and responsibilities of the coordinating centre, steering committee, endpoint adjudication committee, data management team, and other individuals or groups overseeing the trial, if applicable (see Item 21a for data monitoring committee)	-	Pg 15, 31-32
**Introduction**				
Background and rationale	6a	Description of research question and justification for undertaking the trial, including summary of relevant studies (published and unpublished) examining benefits and harms for each intervention	-	Pg 6-7
	6b	Explanation for choice of comparators	-	Pg 12
Objectives	7	Specific objectives or hypotheses	-	Pg 7-8

Trial design	8	Description of trial design including type of trial (eg, parallel group, crossover, factorial, single group), allocation ratio, and framework (eg, superiority, equivalence, noninferiority, exploratory)	-	Pg 8-9
**Methods: Participants, interventions, and outcomes**				
Study setting	9	Description of study settings (eg, community clinic, academic hospital) and list of countries where data will be collected. Reference to where list of study sites can be obtained	-	Pg 10
Eligibility criteria	10	Inclusion and exclusion criteria for participants. If applicable, eligibility criteria for study centres and individuals who will perform the interventions (eg, surgeons, psychotherapists)	-	Pg 10, 16-18, Table S3
Interventions	11a	Interventions for each group with sufficient detail to allow replication, including how and when they will be administered (for specific guidance see TIDieR checklist and guide)	-	Pg 10-12; Figures S2-S3; additional file 2 (completed TIDieR checklist)
	11b	Criteria for discontinuing or modifying allocated interventions for a given trial participant (eg, drug dose change in response to harms, participant request, or improving/worsening disease)	-	Pg 12-13
	11c	Strategies to improve adherence to intervention protocols, and any procedures for monitoring adherence (eg, drug tablet return, laboratory tests)	-	Pg 15-16
	11d	Relevant concomitant care and interventions that are permitted or prohibited during the trial	-	Pg 13
Outcomes	12	Primary, secondary, and other outcomes, including the specific measurement variable (eg, systolic blood pressure), analysis metric (eg, change from baseline, final value, time to event), method of aggregation (eg, median, proportion), and time point for each outcome. Explanation of the clinical relevance of chosen efficacy and harm outcomes is strongly recommended	-	Pg 22-23; Table 1; Figure 3
	12.1		Provide a rationale for the selection of the domain for the trial's primary outcome	Pg 22
	12.2		If the analysis metric for the primary outcome represents within-participant change, define and justify the minimal important change in individuals	Pg 23

	12.3		If the outcome data collected are continuous but will be analyzed as categorical (method of aggregation), specify the cutoff values to be used	Pg 23
	12.4		If outcome assessments will be performed at several time points after randomization, state the time points that will be used for analysis	Pg 9
	12.5		If a composite outcome is used, define all individual components of the composite outcome	Pg 22
Participant timeline	13	Time schedule of enrolment, interventions (including any runins and washouts), assessments, and visits for participants. A schematic diagram is highly recommended (see Figure)	-	Figure 3
Sample size	14	Estimated number of participants needed to achieve study objectives and how it was determined, including clinical and statistical assumptions supporting any sample size calculations	-	Pg 23
	14.1		Define and justify the target difference between treatment groups (eg, the minimal important difference)	Pg 23
Recruitment	15	Strategies for achieving adequate participant enrolment to reach target sample size	-	Pg 18-19
**Methods: Assignment of interventions (for controlled trials)**				
Allocation:				
Sequence generation	16a	Method of generating the allocation sequence (eg, computer-generated random numbers), and list of any factors for stratification. To reduce predictability of a random sequence, details of any planned restriction (eg, blocking) should be provided in a separate document that is unavailable to those who enrol participants or assign interventions	-	Pg 13
Allocation concealment mechanism	16b	Mechanism of implementing the allocation sequence (eg, central telephone; sequentially numbered, opaque, sealed envelopes), describing any steps to conceal the sequence until interventions are assigned	-	Pg 14
Implementation	16c	Who will generate the allocation sequence, who will enrol participants, and who will assign participants to interventions	-	Pg 13-14

Blinding (masking)	17a	Who will be blinded after assignment to interventions (eg, trial participants, care providers, outcome assessors, data analysts), and how	-	Pg 14
	17b	If blinded, circumstances under which unblinding is permissible, and procedure for revealing participants allocated intervention during the trial	-	Pg 14
**Methods: Data collection, management, and analysis**				
Data collection methods	18a	Plans for assessment and collection of outcome, baseline, and other trial data, including any related processes to promote data quality (eg, duplicate measurements, training of assessors) and a description of study instruments (eg, questionnaires, laboratory tests) along with their reliability and validity, if known. Reference to where data collection forms can be found, if not in the protocol	-	Pg 22-23, Table 1
	18a.1		Describe what is known about the responsiveness of the study instruments in a population similar to the study sample	Pg 22
	18a.2		Describe who will assess the outcome (eg, nurse, parent)	Pg 24-26
	18b	Plans to promote participant retention and complete follow-up, including list of any outcome data to be collected for participants who discontinue or deviate from intervention protocols	-	Pg 26
Data management	19	Plans for data entry, coding, security, and storage, including any related processes to promote data quality (eg, double data entry; range checks for data values). Reference to where details of data management procedures can be found, if not in the protocol	-	Pg 26-27
Statistical methods	20a	Statistical methods for analysing primary and secondary outcomes. Reference to where other details of the statistical analysis plan can be found, if not in the protocol	-	Pg 28-29
	20a.1		Describe any planned methods to account for multiplicity in the analysis or interpretation of the primary and secondary outcomes (eg, coprimary outcomes, same outcome assessed at multiple time points, or subgroup analyses of an outcome)	Pg 28-29

	20b	Methods for any additional analyses (eg, subgroup and adjusted analyses)	-	Pg 29
	20c	Definition of analysis population relating to protocol nonadherence (eg, as randomised analysis), and any statistical methods to handle missing data (eg, multiple imputation)	-	Pg 28, 30
**Methods: Monitoring**				
Data monitoring	21a	Composition of data monitoring committee (DMC); summary of its role and reporting structure; statement of whether it is independent from the sponsor and competing interests; and reference to where further details about its charter can be found, if not in the protocol. Alternatively, an explanation of why a DMC is not needed	-	Pg 32
	21b	Description of any interim analyses and stopping guidelines, including who will have access to these interim results and make the final decision to terminate the trial	-	Pg 29, 32
Harms	22	Plans for collecting, assessing, reporting, and managing solicited and spontaneously reported adverse events and other unintended effects of trial interventions or trial conduct	-	Pg 32
Auditing	23	Frequency and procedures for auditing trial conduct, if any, and whether the process will be independent from investigators and the sponsor	-	Pg 32
**Ethics and dissemination**				
Research ethics approval	24	Plans for seeking research ethics committee/institutional review board (REC/IRB) approval	-	Pg 36
Protocol amendments	25	Plans for communicating important protocol modifications (eg, changes to eligibility criteria, outcomes, analyses) to relevant parties (eg, investigators, REC/IRBs, trial participants, trial registries, journals, regulators)	-	Pg 32
Consent or assent	26a	Who will obtain informed consent or assent from potential trial participants or authorised surrogates, and how (see Item 32)	-	Pg 19
	26b	Additional consent provisions for collection and use of participant data and biological specimens in ancillary studies, if applicable	-	Pg 20, 30

Confidentiality	27	How personal information about potential and enrolled participants will be collected, shared, and maintained in order to protect confidentiality before, during, and after the trial	-	Pg 27-28
Declaration of interests	28	Financial and other competing interests for principal investigators for the overall trial and each study site	-	Pg 37
Access to data	29	Statement of who will have access to the final trial dataset, and disclosure of contractual agreements that limit such access for investigators	-	Pg 36
Ancillary and post-trial care	30	Provisions, if any, for ancillary and post-trial care, and for compensation to those who suffer harm from trial participation	-	Pg 13
Dissemination policy	31a	Plans for investigators and sponsor to communicate trial results to participants, healthcare professionals, the public, and other relevant groups (eg, via publication, reporting in results databases, or other data sharing arrangements), including any publication restrictions	-	Pg 33
	31b	Authorship eligibility guidelines and any intended use of professional writers	-	Pg 33
	31c	Plans, if any, for granting public access to the full protocol, participant-level dataset, and statistical code	-	Pg 30
**Appendices**				
Informed consent materials	32	Model consent form and other related documentation given to participants and authorised surrogates	-	https://osf.io/sj9ha/
Biological specimens	33	Plans for collection, laboratory evaluation, and storage of biological specimens for genetic or molecular analysis in the current trial and for future use in ancillary studies, if applicable	-	Pg 28
