# Supplementary material for: Remote monitoring of rheumatoid arthritis (REMORA): study protocol for a stepped wedge cluster randomized trial and process evaluation of an integrated symptom tracking intervention
Source: Trials. 2024 Oct 15;25:683. doi: 10.1186/s13063-024-08497-9 (PMC11481815; doi:10.1186/s13063-024-08497-9)
Supplement: Supplementary file 2 — Additional file 2. Completed TIDieR checklist. [file 13063_2024_8497_MOESM2_ESM.pdf]

Additional file: Completed Template for Intervention Description and Replication (TIDieR) checklist

Item number	Item	Where located ** Primary paper (page or appendix number)	Other † (details)
	**BRIEF NAME**		
**1.**	Provide the name or a phrase that describes the intervention.	6-7; 10-11; reference 38	
	**WHY**		
**2.**	Describe any rationale, theory, or goal of the elements essential to the intervention.	6; Figure S1	______________
	**WHAT**		
**3.**	Materials: Describe any physical or informational materials used in the intervention, including those provided to participants or used in intervention delivery or in training of intervention providers. Provide information on where the materials can be accessed (e.g. online appendix, URL).	10-11; Figures S2-S3; references 38-40	
**4.**	Procedures: Describe each of the procedures, activities, and/or processes used in the intervention, including any enabling or support activities.	10-11	______________
	**WHO PROVIDED**		
**5.**	For each category of intervention provider (e.g. psychologist, nursing assistant), describe their expertise, background and any specific training given.	10, 17; Table S3	______________
	**HOW**		
**6.**	Describe the modes of delivery (e.g. face-to-face or by some other mechanism, such as internet or telephone) of the intervention and whether it was provided individually or in a group.	10-11	______________
	**WHERE**		
**7.**	Describe the type(s) of location(s) where the intervention occurred, including any necessary infrastructure or relevant features.	10-12	______________
	**WHEN and HOW MUCH**		
**8.**	Describe the number of times the intervention was delivered and over what period of time including the number of sessions, their schedule, and their duration, intensity or dose.	11; Table S2	______________
	**TAILORING**		
**9.**	If the intervention was planned to be personalised, titrated or adapted, then describe what, why, when, and how.	11	______________
	**MODIFICATIONS**		
**10.** [b]	If the intervention was modified during the course of the study, describe the changes (what, why, when, and how).	N/A	______________
	**HOW WELL**		
**11.**	Planned: If intervention adherence or fidelity was assessed, describe how and by whom, and if any strategies were used to maintain or improve fidelity, describe them.	15-16; Table 1	https://doi.org/10.21203/rs.3.rs-4712251/v1
**12.**[b]	Actual: If intervention adherence or fidelity was assessed, describe the extent to which the intervention was delivered as planned.	N/A	______________

[a.] N/A indicates the item is not applicable for the intervention being described.

[b.] These items are not relevant to a protocol and cannot be described until the study is complete.
