# Supplementary material for: Remote monitoring of rheumatoid arthritis (REMORA): study protocol for a stepped wedge cluster randomized trial and process evaluation of an integrated symptom tracking intervention
Source: Trials. 2024 Oct 15;25:683. doi: 10.1186/s13063-024-08497-9 (PMC11481815; doi:10.1186/s13063-024-08497-9)

Supplementary material

Tables

Table S1. List of participating hospital sites (i.e., clusters) per region

**Hospital sites in Greater Manchester**
Bolton One Health Centre, Bolton
Fairfield General Hospital, Bury
Rochdale Infirmary, Rochdale
Pennine MSK Partnership, Oldham
Manchester Royal Infirmary, Manchester
Wythenshawe Hospital, Wythenshawe
Stepping Hill Hospital, Stockport
Tameside General Hospital, Ashton-under-Lyne
Trafford General Hospital, Davyhulme
Salford Royal Hospital, Salford
Wrightington Hospital, Wigan
**Hospital sites in North-West London**
Chelsea and Westminster Hospital, London
Central Middlesex Hospital, London
Northwick Park Hospital, Harrow
Hammersmith Hospital, London
St Mary's Hospital, London

Table S2. Instruments, number of items, and scales for REMORA app question sets collected daily, weekly and monthly [a]

**Instrument**	**Number of items**	**Scale**	**Range / anchors**
Daily collection (notifications at 18:30)			
Rheumatoid Arthritis Impact of Disease (RAID) score [b]	7	Visual analogue	0 (lowest severity) to 10 (highest severity)
Morning stiffness	1	7-point Likert	0 min to >2 hours
Weekly collection (notifications at 15:30)			
Swollen Joint Count for 28 joints	1	Numeric rating	0 to 28
Tender Joint Count for 28 joints	1	Numeric rating	0 to 28
Patient global assessment of disease activity	1	Visual analogue	0 (very well) to 10 (very poor)
Work Productivity and Activity Impairment (WPAI) for RA	6	Mix (visual analogue, numeric rating, dichotomous)	0% (lowest impairment; most productive) to 100% (greatest impairment; least productive) [c]
Flare occurrence and description	2	Dichotomous, free text	n/a
Monthly collection (notifications at 12:00)			
Health Assessment Questionnaire (HAQ)	23	Mix (4-point Likert, dichotomous, visual analogue)	0 to 3 (mild/moderate disability) to 3 (severe/very severe disability)

Abbreviations: n/a, not applicable

a. See Figure S2 for how items are presented within the app; interactive screenshots are available on https://marvelapp.com/prototype/6e6ieg7/screens

b. Including pain, function, fatigue, sleep, physical wellbeing, emotional wellbeing, coping

c. Four scores reflecting: work time missed (absenteeism), impairment while working (presenteeism), overall work impairment (absenteeism + presenteeism), activity impairment

Table S3. Inclusion and exclusion criteria per participant type

**Inclusion Criteria**	**Exclusion Criteria**
Healthcare professionals participating in the trial [a]	
• Responsibility for the assessment and care of patients with RA.<br>• Responsibility for treatment decision-making for patients with RA.<br>• Access to the medical record at hospital site as part of routine care.<br>• Willing to review the interactive REMORA dashboard during clinical interactions for consented patients.<br>• Willing to provide full written informed consent.	• No responsibility for the assessment and care of patients with RA.<br>• No responsibility for treatment decision-making for patient with RA.<br>• No access to the medical record at hospital site as part of routine care.<br>• Unwilling to participate in the stepped wedge trial and review the REMORA dashboard during clinical interactions.<br>• Unwilling to provide full written informed consent.
Patients participating in the trial [a]	
• ≥18 years of age (no upper age limit) with active definite or active probable RA, and under the care of a consented healthcare professional. Active disease is defined by meeting *at least one* of the following three criteria:<br>• a DAS28 score of 3.2 or above recorded within six months of the baseline visit or as assessed at the baseline visit (indicative of moderate or high disease activity) [b]<br>• a change in disease-modifying antirheumatic drugs or steroids administered for RA in the last six months.<br>• follow-up visits more frequently than annually, i.e., anticipated to have at least one follow-up visit in the next 12 months from the baseline visit.<br>• Owning or having daily access to an Android or iOS smartphone or tablet with PIN code security, software version that supports the REMORA app, and the ability to access the internet daily to support data upload. The REMORA app will work on the four latest versions available for both Android and Apple.<br>• Regular access to a valid email address.<br>• Having an NHS login account or willing to create one.<br>• Having a medical record accessible via one of two regional data repositories (i.e., *Greater Manchester Care Record* for patients in Greater Manchester region; *Whole Systems and Integrated Care* system for patients in Northwest London).<br>• Able to speak and understand English or supported by someone who can (i.e., if individuals have limited ability to read, speak, write or understand English they must have support from someone who can speak, read and understand English).<br>• Able to follow the requirements of the study independently or with support, including downloading the REMORA app onto a mobile device.<br>• Having capacity and being capable of providing full written informed consent.	• <18 years of age<br>• Not having active definite or active probable RA at the time of recruitment.<br>• Not owning or having daily access to a compatible mobile device.<br>• Not having or unwilling to add PIN code security on the device.<br>• Not having regular access to a valid email address.<br>• Not having or unwilling to create an NHS login account.<br>• Not having a medical record in the *Greater Manchester Care Record* or *Whole Systems and Integrated Care* system<br>• Limited ability to speak and understand English and no support from someone who can.<br>• Not understanding relevant project information (despite support, if needed).<br>• Unable or unwilling to provide full written informed consent.
Eligible healthcare professionals who decline trial participation	
• Invited to take part in the trial and meeting the healthcare professionals' inclusion criteria<br>• Declining trial participation<br>• Willing to be interviewed.<br>• Willing to provide full informed consent either in writing or verbally before or at time of interview.	• Not invited or not meeting the healthcare professionals' inclusion criteria for the trial<br>• Unwilling to be interviewed<br>• Unwilling to provide full written informed consent.

Table S3 (cont.)

Eligible patients who decline trial participation	
• Invited to take part in the trial and meeting the patients' inclusion criteria<br>• Declining trial participation<br>• Willing to be interviewed.<br>• Having capacity and being capable of providing full written informed consent either in writing or verbally before or at time of interview.	• Not invited or not meeting the patients' inclusion criteria for the trial<br>• Unwilling to be interviewed<br>• Unable or unwilling to provide full written informed consent.
Implementers	
• Any professional or volunteer working at a participating hospital site, a collaborating organization, or within the community involved in the technical or organizational implementation of the REMORA2 intervention (i.e., integrated symptom tracking) at a local, regional, or national level.<br>• Willing to be interviewed.<br>• Willing to provide full informed consent either in writing or verbally before or at time of interview.	• Not involved in the implementation of the REMORA2 intervention<br>• Unwilling to participate in an interview.<br>• Unwilling to provide full written informed consent.

Abbreviations: NHS, national health service; RA, rheumatoid arthritis

[a.] Healthcare professionals and patients who are eligible and consented to take part in the trial can also participate in an interview and/or clinic observations as part of the process evaluation, if they are willing and able to provide (separate) full written informed consent for this additional part of the research. However, patient trial participants who took part in the previous REMORA2 feasibility trial (https://doi.org/10.21203/rs.3.rs-4712251/v1) are excluded from the interviews.

[b.] Fransen J, Van Riel PLCM. The Disease Activity Score and the EULAR response criteria. *Clin Exp Rheumatol*. 2005;23:S93-S9.

Table S4. Topics covered in interviews per participant type

**Outcomes assessed** [a]	**Topics covered in interviews** [b]
Healthcare professionals participating in the trial	
• Perceptions of self-management, clinic visits, and decision-making processes<br>• Expectations, experiences and views on the acceptability and usefulness of integrated symptom tracking<br>• Facilitators and barriers to behavior change and intervention implementation and uptake	• Views, perceptions and experiences of integrated symptom tracking, including technical/functional issues, remembering to look at symptom graphs during consultations, and usefulness of the symptom graphs in the interactive REMORA dashboard.<br>• Usefulness of training on how to interpret and use the symptom graphs in the interactive REMORA dashboard<br>• Reasons to (not) prescribe or use integrated symptom tracking, including any barriers to digital inclusion or health equity.<br>• Perceived changes, benefits and disadvantages of using integrated symptom tracking compared with standard of care, including content and process of clinic visits (e.g., time to discuss symptoms, communication with patient, level and quality of (shared) decision-making)
Patients participating in the trial - allocated to integrated symptom tracking [c]	
• Perceptions of self-management, clinic visits, and decision-making processes<br>• Expectations, experiences and views on the acceptability and usefulness of integrated symptom tracking<br>• Facilitators and barriers to behavior change and intervention implementation and uptake	• Views, perceptions and experiences of using health services and health-related apps in general.<br>• Views, perceptions and experiences of tracking and self-management of symptoms, and the process, communication and decision-making during clinic visits in general.<br>• Usefulness of instructions to download, setup and use the REMORA app.<br>• Views, perceptions and experiences of integrated symptom tracking, including views on the REMORA app's functionality, ease of use, and usefulness of in-app symptom graphs.<br>• Reasons to (not) use integrated symptom tracking, including any barriers to digital inclusion and health equity.<br>• Perceived changes, benefits and disadvantages of using integrated symptom tracking compared with standard of care, including quality of life and wellbeing, self-management, and content and process of clinic visits (e.g., communication with the care team, level and quality of (shared) decision-making).
Patients participating in the trial - allocated to standard of care	
• Perceptions of self-management, clinic visits, and decision-making processes<br>• Expectations and views on the acceptability and usefulness of integrated symptom tracking<br>• Facilitators and barriers to behavior change and intervention implementation and uptake	• Views, perceptions and experiences of using health services and health-related apps in general.<br>• Views, perceptions and experiences of tracking and self-management of symptoms, and the process, communication and decision-making during clinic visits in general.
Eligible healthcare professionals who decline trial participation	
• Expectations and views on the acceptability and usefulness of integrated symptom tracking<br>• Facilitators and barriers to behavior change and intervention implementation and uptake	• Reasons and rationale for declining trial participation and barriers to participation in future trials evaluating similar/related digital health innovations<br>• Perceived barriers to using integrated symptom tracking in clinical practice

Table 4. (cont.)

Eligible patients who decline trial participation	
• Expectations and views on the acceptability and usefulness of integrated symptom tracking<br>• Facilitators and barriers to behavior change and intervention implementation and uptake	• Reasons and rationale for declining trial participation and barriers to participation in future trials evaluating similar/related digital health innovations<br>• Views on the participant-facing materials explaining the trial<br>• Perceived barriers to digital inclusion and health equity, and what additional support may be needed to address these<br>• Personal care arrangements (e.g., how health and care status impacts on ability to participate).<br>• Views, perceptions and experiences of accessing and using health services and health-related apps in general, including using mobile devices to collect health data to support clinical care
Implementers	
• Facilitators and barriers to behavior change and intervention implementation and uptake	• Views, perceptions and experiences of the process to implement the REMORA2 intervention<br>• Views on need for and potential benefits of integrated symptom tracking<br>• Challenges to implement integrated symptom tracking across organizational boundaries and how to address these<br>• Resources required to implement integrated symptom tracking, including costs for setup and maintenance.<br>• Local organizational factors that influenced implementation.<br>• Adjustments and possible education needed for successful future implementations of integrated symptom tracking

a. See Table 1 in main text
b. We will iteratively refine topics guides based on the (preliminary findings from the) interviews we conduct throughout the process evaluation
c. Interviews with patient trial participants who are allocated to integrated symptom tracking but do not submit any symptom report (i.e., do not start symptom tracking) also cover some of the topics explored in the interviews with eligible patients who decline trial participation.

Figures

Figure S1: Logic model showing our hypothesis of how the REMORA2 intervention (i.e., integrated symptom tracking) works

1. Problem	2. Assumptions	3. Intervention targets	4. Intervention activities	5. Mechanisms of change	6. Outcomes [a]
Poor patient recall of symptoms and flares since last clinic visit<br><br>Incomplete and inaccurate information on patients' disease activity<br><br>Suboptimal treatment decision-making<br><br>Worse patient experience and outcomes	Patients own or have access to a mobile device<br><br>Patients are willing and able to use the mobile device for tracking and sharing their symptoms<br><br>Healthcare professionals can access the symptom data from the electronic health record system without having to log into a separate system<br><br>Healthcare professionals have time and are willing to review and discuss the symptom data in clinic	The symptom data provides new insights (e.g., into response to treatment, or into frequency, time and duration of flares)<br><br>Patients can interpret the tracked symptom data and use it to change their self-management behavior<br><br>Healthcare professionals can interpret the tracked symptom data and use it inform treatment decisions<br><br>Patients and healthcare professionals reviewing/discussing the tracked symptom data in clinic facilitates shared decision-making	Materials and support to help patients set up and use the REMORA symptom tracking app<br><br>Patients' daily tracking of symptoms and weekly/monthly tracking of other aspects of RA<br><br>Automated in-app reminders for patients to track<br><br>Interactive graphs to visualize the symptom data in the REMORA app<br><br>Daily diary function in REMORA app for patients to keep notes on their RA (not shared with healthcare professionals)<br><br>Interactive REMORA dashboard in the electronic health record to visualize the symptom data for healthcare professionals<br><br>Training for healthcare professionals to use the interactive dashboard and discerning patterns of time-varying disease activity	**Patients**<br>Engagement with integrated symptom tracking<br><br>Feeling empowered (not burdened) by symptom tracking<br><br>Insight into own disease activity (e.g., how it fluctuates over time, what influences it)<br><br>Change in health behavior and self-management<br><br>Improved communication with healthcare professionals<br><br>**Healthcare professionals**<br>Engagement with integrated symptom tracking<br><br>Symptom data in clinic adds value (not burden)<br><br>Better understanding patient contexts and priorities<br><br>Feeling enabled and encouraged to involve patients in treatment decisions<br><br>Improved communication with patients<br><br>Change in treatment decisions	**Primary outcome**<br>Decreased clinician-reported disease activity<br><br>**Secondary outcomes**<br>Decreased patient-reported disease activity<br><br>Improved health-related quality of life<br><br>Decreased work impairment<br><br>Decreased disability<br><br>More optimal medication and resource use<br><br>Improved shared decision-making<br><br>Improved experience of clinic visits (patient enablement)<br><br>Improved self-management behavior<br><br>Improved patient understanding of RA

Abbreviations: REMORA, REmote MOnitoring of Rheumatoid Arthritis; RA, rheumatoid arthritis

a. See Table 1 in the manuscript for how we assess each outcome

Figure S2: Screenshots from REMORA smartphone app home screen and from example daily, weekly, and monthly questions *

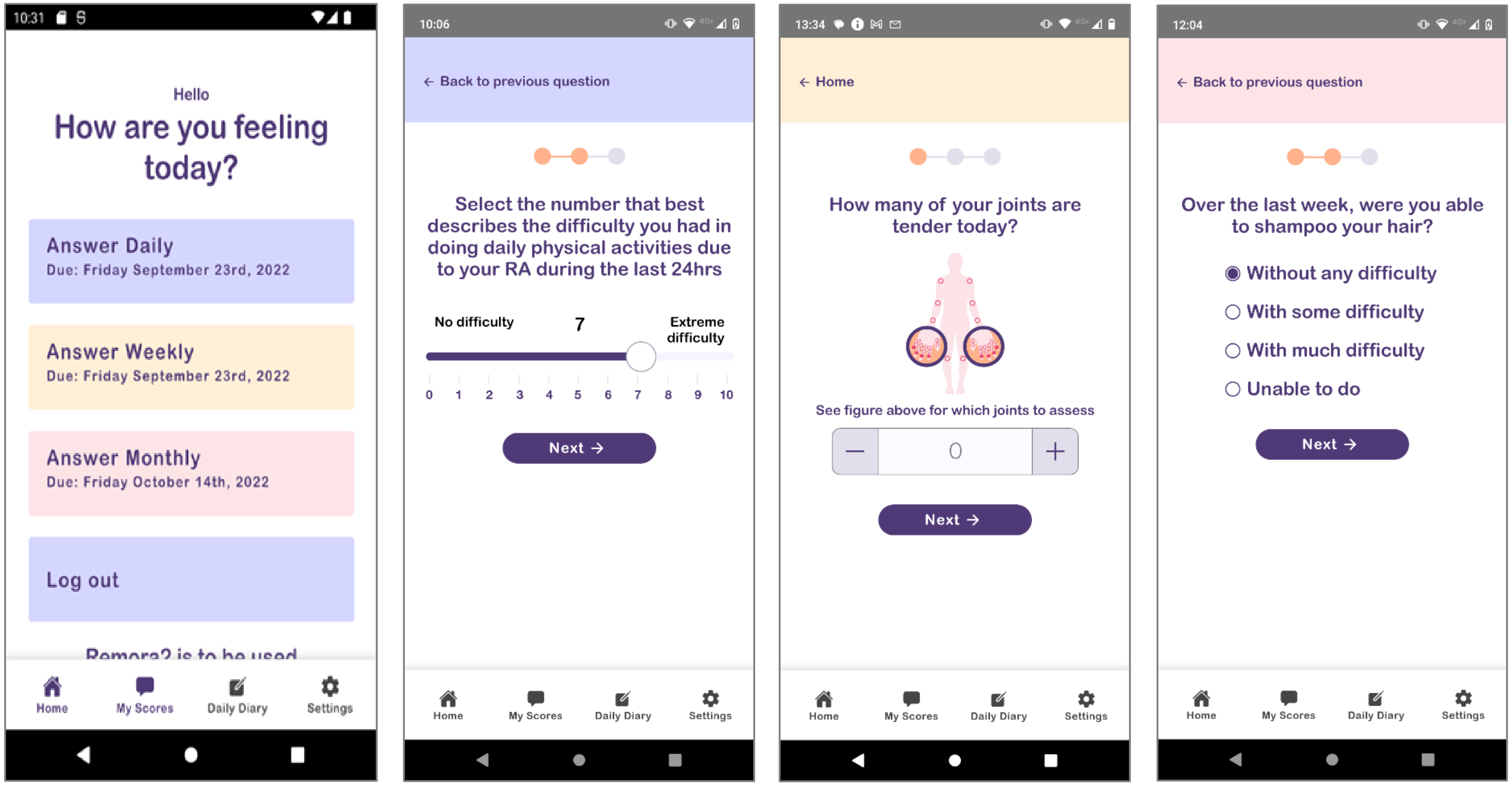

Home screen | *Example daily question* | *Example weekly question* | *Example monthly question*

* *Interactive screenshots are available on https://marvelapp.com/prototype/6e6ieg7/screens and via a public repository on https://doi.org/10.48420/26662249*

Figure S3: Screenshots from interactive REMORA dashboard integrated in hospital sites' electronic health record system with daily (top panel) and weekly/monthly (bottom panel) patient-reported app data *

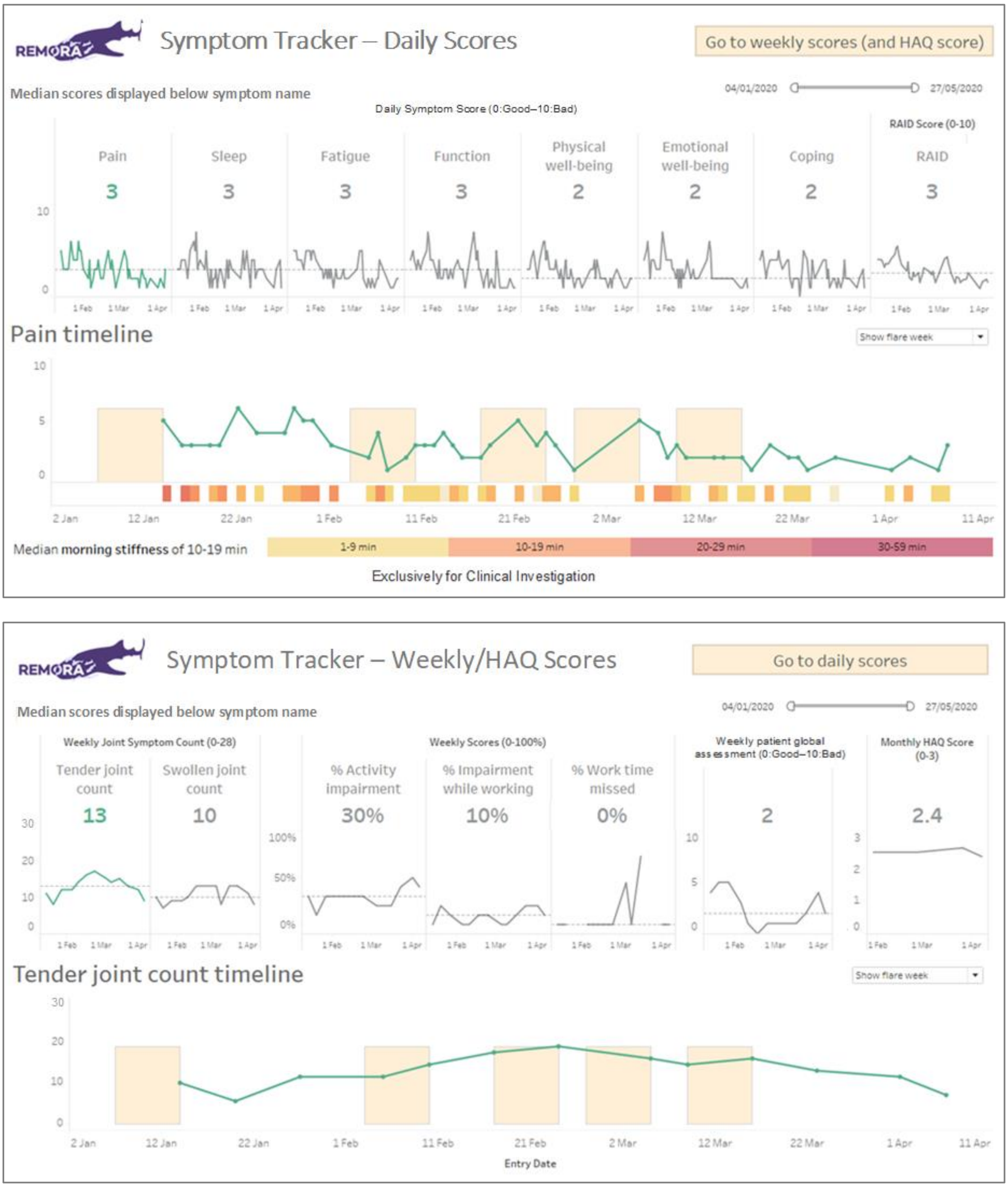

** The bars in the graphs displayed in the bottom half of the screens show in which weeks patients reported a flare (e.g., the week preceding 12-Jan in the above screenshots). As users of the dashboard, healthcare professionals can switch these 'flare bars' on or off in line with their preference.*

Figure S4: REMORA-branded materials to improve intervention uptake and adherence (for example, lanyards and pin badge that healthcare professionals can wear to help patient participants mention their involvement in the trial during clinic visits)

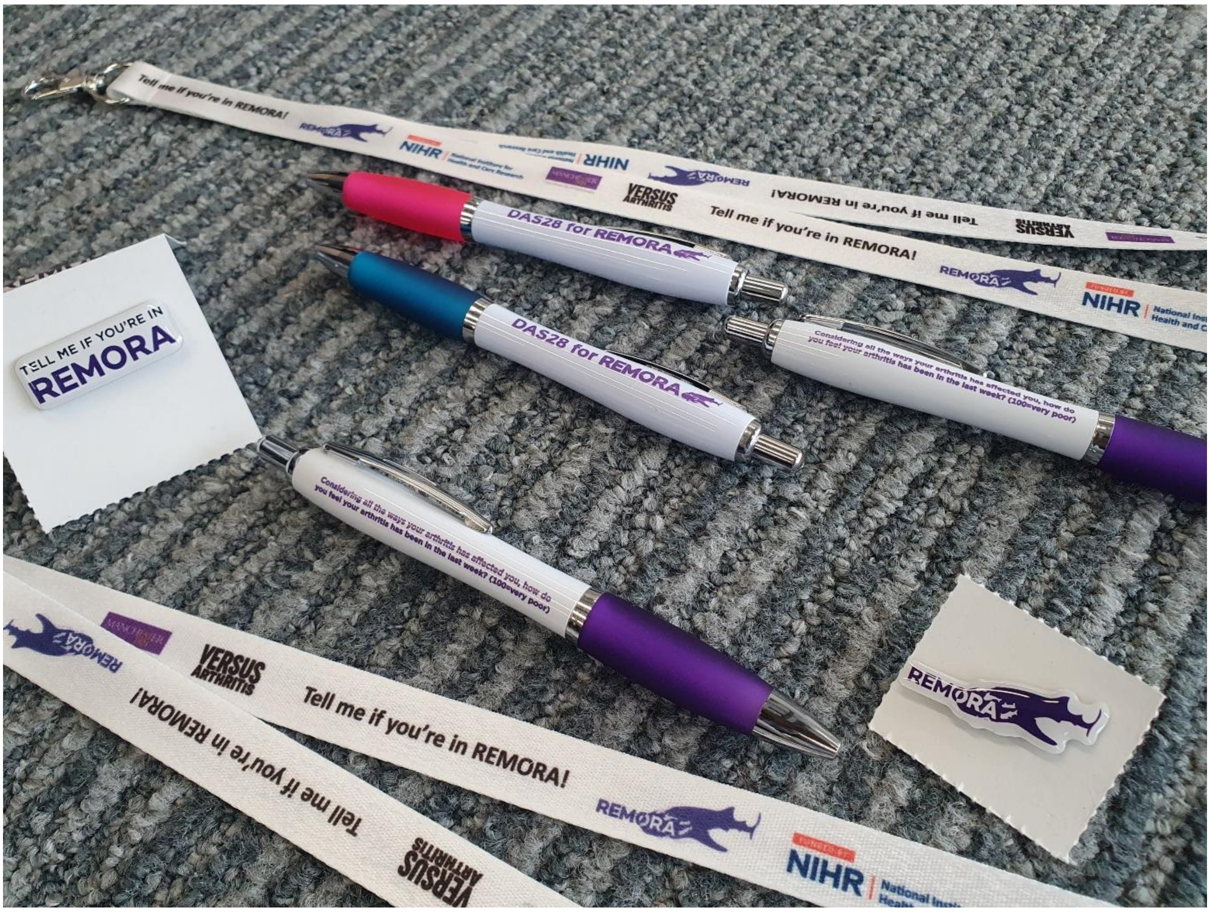

Supplement: Supplementary file 3 — Additional file 3. Supplementary materials. [file 13063_2024_8497_MOESM3_ESM.pdf]
